# Distinct domains of LINGO1 control surface expression and biophysical properties of large conductance Ca^2+^- and voltage-activated potassium (BK) channels

**DOI:** 10.1016/j.jbc.2025.110550

**Published:** 2025-08-05

**Authors:** Yaly Al Kawadri, Heather McClafferty, Srikanth Dudem, Kaneez E. Rabab, Keith D. Thornbury, Gerard P. Sergeant, Mark A. Hollywood, Michael J. Shipston

**Affiliations:** 1 Smooth Muscle Research Centre, Dundalk Institute of Technology, Louth, Ireland; 2 Centre for Discovery Brain Sciences, Edinburgh Medical School: Biomedical Sciences, University of Edinburgh, Scotland

**Keywords:** potassium channel, LINGO subunits, electrophysiology, surface expression, leucine-rich repeat–containing proteins

## Abstract

Large conductance Ca^2+^ and voltage-activated K^+^ (BK) channels are ubiquitous ion channels that regulate a wide array of physiological process. Their functional diversity can be modulated by accessory proteins including a family of leucine-rich repeat and immunoglobulin-like domain–containing protein (LINGO) subunits that may control both the biophysical properties and surface trafficking of BK channels. By exploiting differences in the regulation of BK channels by LINGO1 and LINGO2 subunits we have taken a chimeric approach to dissect the role of distinct domains of these single transmembrane pass proteins. We demonstrate that the large extracellular domain of LINGO1, but not the transmembrane or intracellular C-terminal tail domain (ITD), is required to inhibit plasmalemmal expression of BK channels. Importantly, this inhibitory effect did not require the 12 leucine-rich repeats) in the extracellular domain. In contrast, the ITD is important for controlling the shift in voltage of half maximal activation of BK:LINGO currents seen between LINGO1 and LINGO2 at different Ca^2+^ concentrations. This effect on voltage of half maximal activation is independent of the contribution of the last 8 amino acids of the ITD, which confer inactivation on BK:LINGO currents. These data reveal that distinct domains of LINGO proteins are critical determinants of the function and properties of BK channels.

BK channels regulate a diverse array of physiological processes including control of neuronal excitability, smooth muscle contraction, epithelial ion transport, and endocrine hormone release (1, 2, 3, 4, 5). Dysfunction of BK channels is thus associated with a variety of neurological, cardiovascular, and endocrine/metabolic disorders (1, 2, 3, 4, 5). The different physiological functions of BK channels, encoded by a single pore-forming α-subunit (KCNMA1) in distinct tissues, results from a variety of post-transcriptional and post-translational regulatory mechanisms including alternative splicing, assembly with regulatory subunits, and diverse modes of post-translational modification (3, 6, 7, 8). The pore of the BK channel is formed by the tetrameric assembly of BKα-subunits that can associate with a variety of regulatory β_1-4_, γ _1-4_, and LINGO_1-4_ subunits (3, 9, 10). Structurally, LINGO subunits, like the γ _1-4_ subunits, are leucine-rich repeat (LRR)-containing (LRRC) proteins with a single transmembrane helix and a short intracellular C-terminal tail. However, in contrast to the γ-subunits, LINGO1 contains 12 rather than 6 LRR domains in the N-terminal extracellular domain (ED) and possesses an Ig1-like domain that is absent in the γ-subunits (11, 12, 13).

Functionally, co-expression of BK channels with LINGO1 and LINGO2 subunits results in rapidly inactivating currents. Furthermore, in 100 nM Ca^2+^ BK channels containing LINGO1 (BK:LINGO1) or LINGO2 (BK:LINGO2) activate at more negative membrane potentials than BKα alone (9, 10). Although LINGO1 and LINGO2 share 61% identity and 77% homology (14, 15), there are significant differences in their effects on both the biophysical and cell surface expression of BK channels. First, coexpression of BKα with LINGO1 (BK:LINGO1) reduced plasmalemmal expression of BK channels, whereas LINGO2 co-expression with BKα failed to alter this (9, 10). Second, when currents are recorded under 100 nM intracellular free Ca^2+^ conditions, BK:LINGO1 caused a larger leftward shift in the voltage for half-maximal activation (V_1/2ACT_) than BK:LINGO2 (9, 10). Finally, BK:LINGO1 currents inactivate more rapidly than BK:LINGO2 currents (9, 10).

Although the intracellular C-terminal tail domain (ITD) of LINGO1 and LINGO2 are important for inactivation of BK channels (9, 10), the contribution of the transmembrane domain (TMD) or large ED to the biophysical properties and plasmalemmal expression of BK channels is unknown. We reasoned that a chimeric domain-swap approach would allow us to interrogate the role of the ED, TMD, or ITD in the control of BK channel properties.

Our studies reveal that the ED domain of LINGO1 is a critical determinant of the LINGO1-mediated suppression of plasmalemmal expression of BK channels, whereas the ITD domain appears most likely to contribute to the negative shift in V_1/2ACT_ in LINGO1 and LINGO2, respectively.

## Results

**Figure 1:**
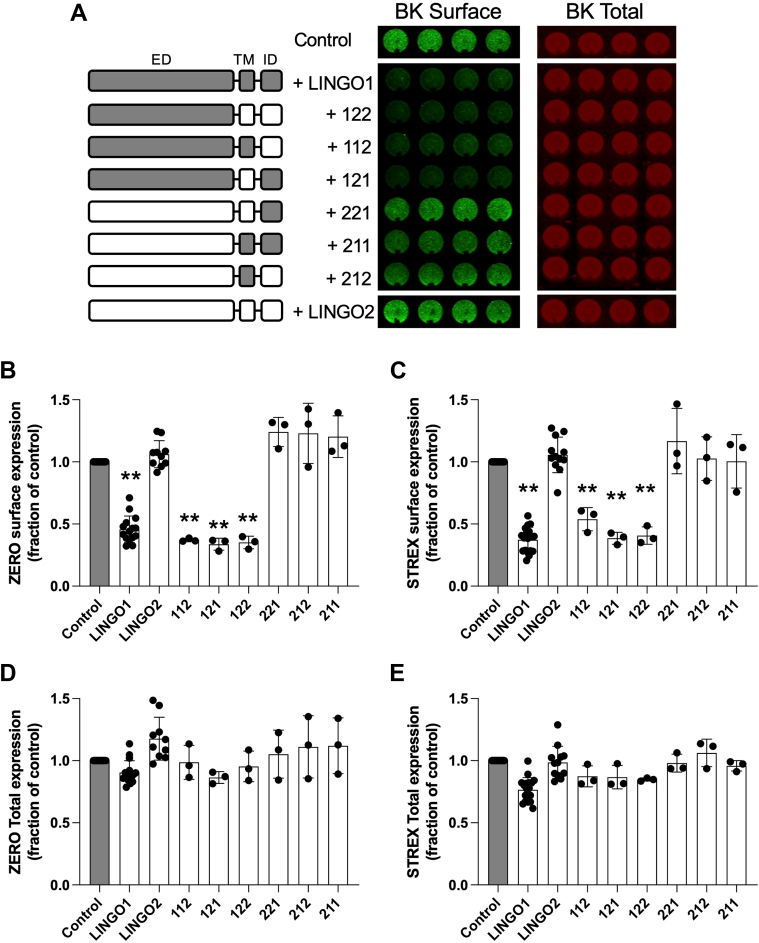
**Extracellular domain of LINGO1 controls cell surface expression of BK channels.***A*, schematic of LINGO chimeras and representative on-cell Western assay. Chimera nomenclature is based on extracellular domain (ED), transmembrane domain (TMD), and intracellular tail domain (ITD) from LINGO1 or LINGO2, respectively. Representative experiment (*right hand panels*) from an on-cell Western assay to detect cell surface expression of the ZERO splice variant of BK channels in HEK293 cells in the presence of different LINGO chimeras, run in quadruplicate. BK surface expression (Flag-) was determined in nonpermeabilized cells probing for the extracellular Flag-epitope on the BK channel extracellular N terminus. Total BK expression was determined in the same well after cell permeabilization and probing for the -myc epitope on the intracellular C terminus of the BK channel. *B* and *C*, quantification of BK channel surface expression for ZERO and STREX splice variant of BK channel, expressed as a fraction of the Flag/myc ratio in the absence of LINGO1. *D* and *E*, corresponding total BK channel expression expressed as a fraction of total BK channel in absence of LINGO1. Data are mean ± SD from 3 to 20 independent experiments in each group. ∗*p* < 0.05, ∗∗*p* < 0.01 *versus* BK alone Kruskal–Wallis with Dunn’s *post hoc* test. LINGO, leucine-rich repeat and immunoglobulin-like domain–containing protein.

To investigate the contribution of different domains of LINGO1 in the control of plasmalemmal surface expression of BK channels, we used an on-cell Western (OCW) approach employed previously (10) to demonstrate suppression of BKα surface expression by LINGO1 (Fig. 1). The mouse ZERO variant of BKα (referred to as BK) with an extracellular Flag-tag (to determine surface channel expression) and intracellular Myc-tag (to determine total channel expression) was coexpressed with Flag-tagged LINGO1, LINGO2, or domain-swap chimeras in HEK293 cells in a ratio of 0.5 μg LINGO to 1 μg BK. Domain-swap chimeras are identified using a 3 digit nomenclature with 1 indicating a domain from LINGO1 and 2 denoting a domain from LINGO2. The domains are in order of ED, TMD, and ITD (Figs. 1 and S1). For example, in this nomenclature LINGO1 would be 111, LINGO2 would be 222 and a chimera where the ED domain of LINGO2 replaced the ED domain of LINGO1 in a LINGO1 background would be 211.

### The ED of LINGO1 controls surface expression of BKα-subunits

As reported previously (9, 10), co-expression of BK with LINGO1 resulted in a significant suppression of cell surface BK channels (Fig. 1, *A* and *B*). In contrast, co-expression of LINGO2 with BK had no significant effect on cell surface or total channel expression (Fig. 1*C*). We confirmed that the differential effect of LINGO1 to suppress cell surface expression of BK was not simply a dose-dependent effect of LINGO1 and LINGO2 expression (Fig. S2), in particular, as total LINGO2 expression was consistently lower in HEK293 cells than LINGO1 (Fig. S3). We titrated different ratios of LINGO1 or LINGO2 complementary DNA (cDNA) with a fixed amount of BK cDNA (Fig. S2). While LINGO1 concentration-dependently suppressed BK surface expression, LINGO2 had no significant effect even at a 10-fold excess compared to LINGO1.

For all LINGO chimeras containing the ED of LINGO1, irrespective of the TMD or ITD configuration (*i.e.* chimeras 112, 121, 122), co-expression with BK resulted in a significant suppression of BK surface expression that was indistinguishable from WT LINGO1 (Fig. 1, *A* and *B*). In contrast, for all LINGO chimeras expressing the ED of LINGO2 (*i.e.* chimeras 221, 211, 212), no significant suppression of BK surface expression was observed, as for WT LINGO2 (Fig. 1*B*). The lack of effect of LINGO2 chimeras was not a result of lower expression levels of these constructs as all LINGO2 chimeras expressed at the same level as WT LINGO1 (Fig. S3). The suppression of BK surface expression was also not a result of reduced total expression of the BKα-subunit upon coexpression with any chimera (Fig. 1*D*). This suggests that the ED of LINGO1 is the key determinant controlling surface expression of BK with the TMD or ITD of LINGO1 having little impact. To examine if the inhibitory effect of LINGO1 depended on the level of BK channel surface expression *per se,* we next tested if LINGO1 could also inhibit surface expression of the BKα splice variant STREX. This variant possesses a 59 amino acid insert in the intracellular C terminus of BKα and has been shown to express ∼2-fold higher at the cell surface than the BK ZERO splice variant in HEK293 cells ((16) and Fig. S4*B*). As observed with the BK ZERO splice variant, coexpression of the STREX splice variant with any LINGO chimera containing the ED of LINGO1 (LINGO1 and chimeras 112, 121, 122) resulted in a significant suppression of STREX surface expression similar to that observed with ZERO BKα-subunits (Fig. 1*C*). LINGO chimeras with the LINGO2 ED also failed to alter STREX surface expression significantly (Figs. 1, *C* and *D* and S2*B*). Furthermore, LINGO1 had no significant effect on internalization of either BKα variant (Fig. S4), suggesting that the major effect of the LINGO1 ED was to suppress forward trafficking of the BKα-subunit to the plasma membrane.

**Figure 2:**
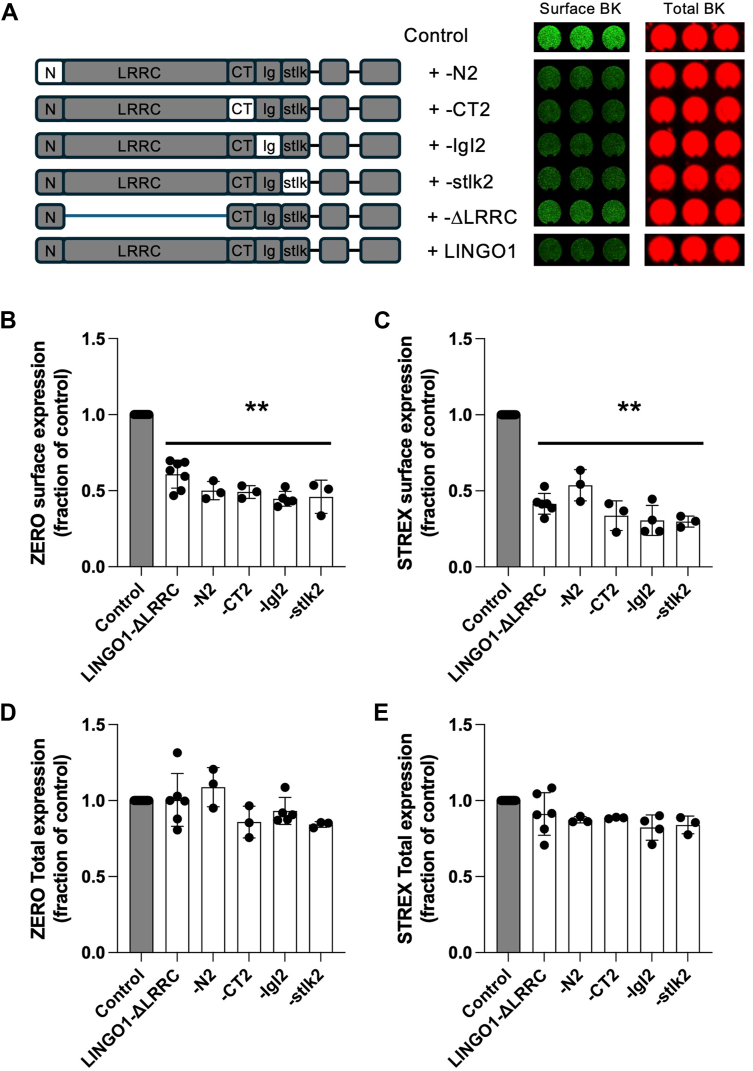
**LRRC repeat subdomain of LINGO1 extracellular domain is not required for LINGO1 suppression of BK channel surface expression.***A*, schematic of LINGO chimeras with LRRC repeat deletion (ΔLRRC) and subdomain swaps in the ED of LINGO1 with corresponding LINGO2 subdomains: N-terminus (N-), C-terminal LRR-Ct (-Ct), Ig1-like domain (Ig1), and stalk domain (-stalk). Representative experiment (*right hand panels*) from an on-cell Western assay to detect cell surface expression of the ZERO splice variant of BK channels in HEK293 cells in the presence of different LINGO chimeras as in Figure 1. *B* and *C*, quantification of epitope-tagged Flag-ZERO-myc and Flag-STREX-myc splice variant of BK channel surface expression with different LINGO1 ED subdomain chimeras, expressed as a fraction of the Flag/myc ratio in the absence of LINGO1. *D* and *E*, corresponding total BK channel expression expressed as a fraction of total BK channel in absence of LINGO1. Data are mean ± SD from 3 to 8 independent experiments in each group. ∗*p* < 0.05, ∗∗*p* < 0.01 *versus* BK alone Kruskal–Wallis with *post hoc* Dunn’s test. ED, extracellular domain; LINGO, leucine-rich repeat and immunoglobulin-like domain–containing protein; LRRC, leucine-rich repeat containing.

Since the ED, but not the TMD or ITD of LINGO1 was critical for controlling plasmalemmal expression of coexpressed BKα-subunits, we next examined if distinct subdomains of the LINGO1 ED were important for this suppression. The ED of LINGO1 is composed of 12 LRRs comprising the LRRC domain, a short N-terminal domain (N1), a C-terminal LRRC domain (CT), an IgG-like domain (IgI1) and a “stalk” domain immediately adjacent to the extracellular end of the TMD (Figs. 2 and S1). We thus engineered a series of LINGO1 chimeras in which the corresponding subdomain region of LINGO2 individually replaced the LINGO1 ED subdomain in the LINGO1 background. We also engineered a LINGO1 ΔLRRC mutant in which the LRRC repeat subdomain was removed so that N1 was directly fused to CT (Figs. 2*A* and S5). All these chimeras expressed at similar levels to WT LINGO1 apart from LINGO1 ΔLRRC whose expression was reduced similar to that of LINGO2 (Fig. S3*B*).

Co-expression of the LINGO1 ΔLRRC mutant with either BK ZERO or STREX α-subunits resulted in a significant suppression of plasmalemmal BKα-subunit expression which was indistinguishable from WT LINGO1 (Fig. 2, *A*–*C*). Importantly, co-expression of BK with the LINGO1 ΔLRRC construct produced rapidly inactivating BK currents (Fig. S5). These BK:LINGO1 ΔLRRC currents had an V_1/2ACT_ of 113 ± 9 mV, (n = 9, in 100 nM Ca^2+^) which was similar to that of full-length LINGO1 (118 ± 9 mV, n = 11). These data demonstrate that deletion of the LRRC domains, while reducing total LINGO1 ΔLRRC expression, did not alter functional expression or assembly of LINGO1 ΔLRRC with BK. Furthermore, in chimeras with individual subdomain swaps of LINGO1 for LINGO2, co-expression of ZERO or STREX BKα-subunits resulted in a similar suppression of plasmalemmal BKα-subunit expression. We performed the converse experiment to determine if individual subdomains of the LINGO1 ED could confer inhibition of cell surface expression of BKα-subunits to LINGO2 (Fig. S6). We thus engineered chimeras with individual subdomain swaps of LINGO1 ED subdomains into the LINGO2 background. However, no subdomain-swap chimera conferred the inhibitory phenotype onto LINGO2 for cell surface expression of co-expressed ZERO or STREX BKα-subunits (Fig. S6, *A*–*E*). Interestingly, these subdomain swaps expressed at higher levels than WT LINGO2 and were similar to WT LINGO1 (Fig. S3), except for the stlk1 chimera. Taken together, while these data revealed that the LRRC repeat subdomain of LINGO1 ED was not required for suppression of BKα-subunit surface expression, other individual subdomains of the LINGO1 ED alone were not responsible for the inhibitory effect on BKα-subunit plasmalemmal expression. Rather, it is likely that multiple structural features of the ED domain that did not require LRRC subdomain were important for the suppression of BKα-subunit surface expression. To further test this, we constructed additional domain-swap chimeras in the LINGO1 ΔLRRC background (Fig. S7, *A* and *B*). Swapping either the LINGO2-CT2 or LINGO2-Ig2 subdomains for the corresponding LINGO1 subdomains in LINGO1 ΔLRRC effectively abolished the inhibitory effect on ZERO variant BK channel surface expression. Both LINGO1 ΔLRRC-CT2 and LINGO1 ΔLRRC-Ig2 chimeras expressed at identical levels to LINGO1 ΔLRRC (Fig. S7*D*).

### All LINGO chimeras produce inactivating currents when coexpressed with BK channels

**Figure 3:**
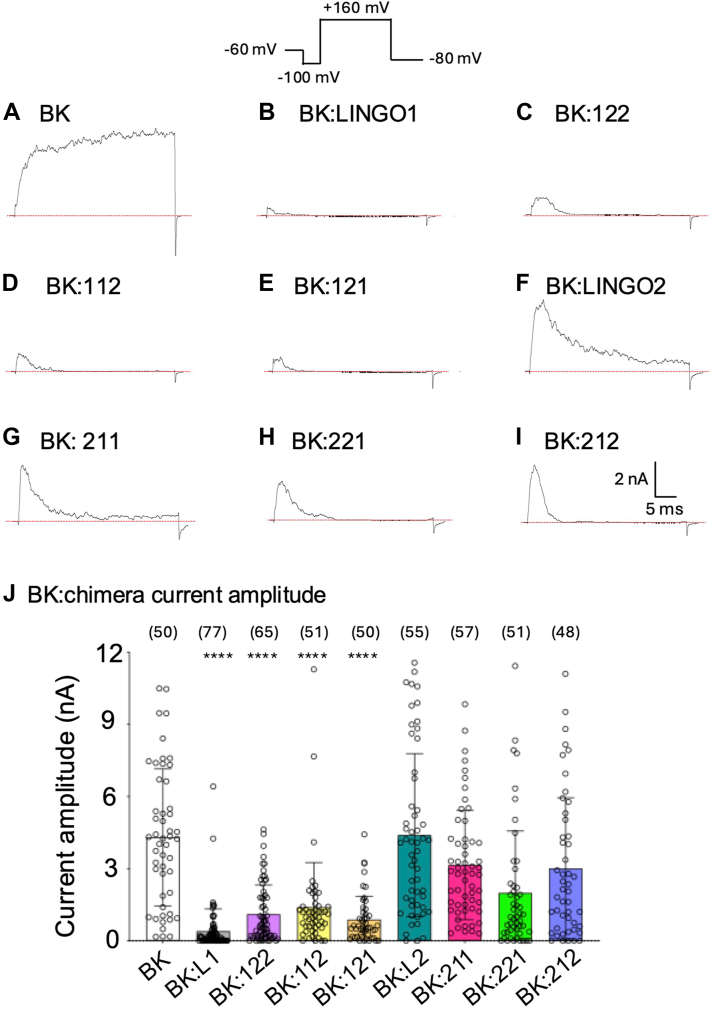
**Inactivating currents of BK, BK:LINGO1, BK:LINGO2, and their BK:chimeric constructs**. All patches were obtained with 5 MΩ pipettes and currents were elicited by stepping to +160 mV for 40 ms, with 100 nM Ca^2+^ bathing the cytosolic surface of the patches. Tail currents were evoked at −80 mV. *A*–*I*, show traces for BK, BK:LINGO1, BK:LINGO2, and their chimeras. *J*, shows the mean current amplitude for BK, BK:LINGO1, BK:LINGO2, and their chimeras. Data are mean ± SD from 48 to 77 independent patches in each group. ∗∗∗∗*p* < 0.0001 compared to BK alone, one-way ANOVA, Tukey’s multiple comparison. LINGO, leucine-rich repeat and immunoglobulin-like domain–containing protein.

To confirm if the suppressive effect of the LINGO1 ED domain also reduced BK:LINGO1 ionic currents, we performed patch clamp electrophysiology using the ZERO variant of BKα and the LINGO1 chimeras expressed in HEK293 cells. As previously reported (9, 10) co-expression of LINGO1 with BKα resulted in inactivating currents with a significantly attenuated peak maximal current, compared to the steady-state large amplitude currents evoked upon step depolarization for BKα expressed alone (Fig. 3, *A*, *B* and *J*). In accordance with the OCW data, co-expression of BKα with LINGO chimeras containing the ED domain of LINGO1 (chimeras 112, 121, 122) also resulted in inactivating currents (Fig. S8) with significantly attenuated peak current amplitude, as for WT LINGO1 (Fig. 3, *C*, *D*, *E*, and *J*). The mean peak current amplitude for BKα alone was 4304 ± 2849 pA (n = 50). Co-expression with LINGO1 caused a ∼93% reduction in current amplitude (401 ± 923 pA, n = 77, *p* < 0.0001). A significant reduction in current amplitude was noted for BK:122 (1107 ± 1212 pA, n = 65), BK:112 (1398 ± 1858 pA, n = 51), and BK121 (884 ± 972 pA, n = 50), when compared to BK alone (*p* < 0.0001). In contrast, while co-expression of LINGO2 also conferred inactivation on BKα currents (Fig. S9), it did not suppress peak current (for BK:LINGO2 4395 ± 3390 pA, n = 55, Fig. 3, *F* and *J*) consistent with previous studies (9). In addition, while chimeras with the ED of LINGO2 (211, 212) all displayed inactivation (Fig. S9), they did not significantly suppress maximal peak current amplitude of BKα currents (Fig. 3, *G*, *H*, and *J*). However, currents from BK:221 had significantly smaller current amplitudes (1993 ± 2575 pA, n = 51, *p* < 0.05) than BK:LINGO2 (Fig. 3, *H* and *J*) and this effect may be due to a difference in its V_1/2ACT,_ as shown later. Thus, the ED of LINGO1 is required for suppression of BKα currents, consistent with the observations from the OCW assays.

### The ITD domain is an important determinant V_1/2ACT_ of BK:LINGO currents

**Figure 4:**
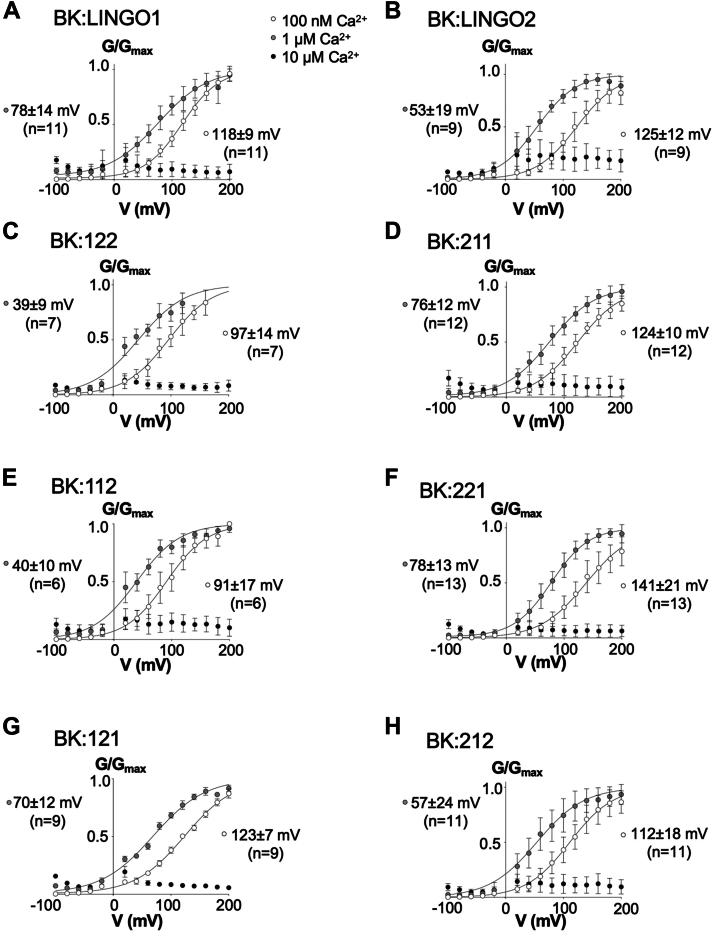
**GV curves for BK:LINGO1, BK:LINGO2, and BK:LINGO chimeras.***A*–*H*, shows GV curve summaries in inside-out configuration fitted with Boltzmann equation (*black lines*) for BK:LINGO1, BK:LINGO2, BK:122, BK:211, BK:112, BK:221, BK:121, and BK:212 in 100 nM Ca^2+^(*white circles*), 1 μM Ca^2+^ (*gray circles*) and 10 μM Ca^2+^(*black circles*), respectively. Data are mean ± SD from 6 to 13 independent experiments in each group. LINGO, leucine-rich repeat and immunoglobulin-like domain–containing protein.

**Figure 5:**
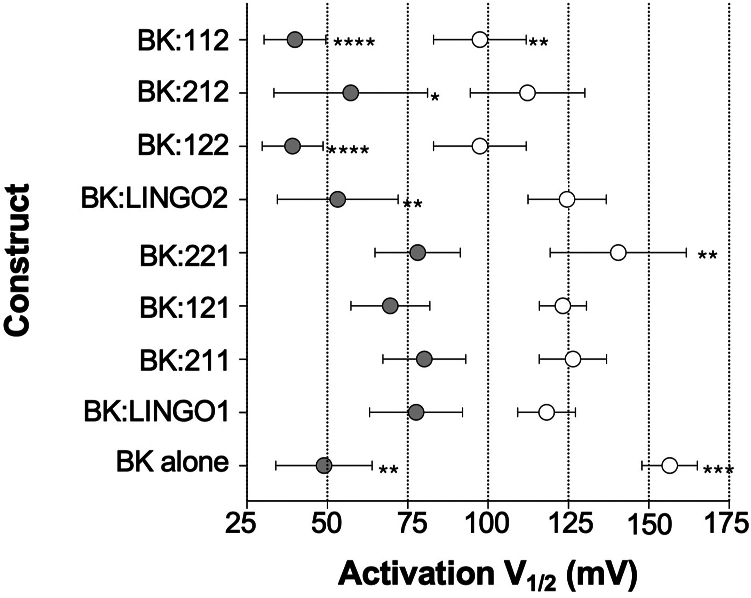
**LINGO C-terminal tail domain contributes to the voltage of half maximal activation of BK:LINGO currents in 1 μM Ca^2+^**. Summary of voltage of half maximal activation (V_1/2ACT_) between LINGO1 and the chimeras in 100 nM and 1 μM Ca^2+^. Data are mean ± SD from 6 to 13 independent experiments in each group. ∗*p* < 0.05, ∗∗*p* < 0.01, ∗∗∗*p* < 0.001, and ∗∗∗∗*p* < 0.0001 compared to the respective *V*_*1/2ACT*_ for BK:LINGO1 in either the presence of 100 nM or 1 μM Ca^2^, one-way ANOVA, Dunnett’s multiple comparison test. LINGO, leucine-rich repeat and immunoglobulin-like domain–containing protein.

As previously reported, in 100 nM free Ca^2+^, both BK:LINGO1 and BK:LINGO2 currents display a V_1/2ACT_ that is more negative than BKα currents alone (9, 10). We thus exploited the LINGO chimeras to identify the domains controlling the negative shift in V_1/2ACT_ by constructing G/G_MAX_ curves under 100 nM or 1 μM free [Ca^2+^] conditions (Fig. 4) and plotting the V_1/2ACT_ compared to the WT LINGO, respectively, for each chimera (Fig. 5).

In the presence of 100 nM Ca^2+^, co-expression of BKα with either LINGO1 or LINGO2 resulted in a significant left shift in the V_1/2ACT_ of BK:LINGO currents (BK:LINGO1 was 118 ± 9 mV, n = 11; BK:LINGO2 was 125 ± 12 mV, n = 9, Fig. 4, *A* and *B*) compared to BKα currents alone (157 ± 9 mV, n = 6, Fig. 5). In the presence of 1 μM Ca^2+^, the V_1/2ACT_ for BK currents alone was 49 ± 15 mV (n = 6) and coexpression with LINGO1 resulted in BK:LINGO1 currents with a significantly right shifted V_1/2ACT_ compared to BK alone of 78 ± 14 mV (n = 11). In contrast, coexpression with LINGO2 resulted in BK:LINGO2 currents with a V_1/2ACT_ (53 ± 19 mV, n = 9) similar to that of BK currents alone (Figs. 4*B* and 5).

The V_1/2ACT_ of chimeras 211 and 121 with a LINGO1 ITD (BK:211, 124 ± 10 mV; BK:121, 123 ± 7 mV, n = 9) were similar to BK:LINGO1 in 100 nM Ca^2+^ (118 ± 9 mV, n = 11, *p* ≥ 0.94). Although the BK:221 currents showed a right shift to 141 ± 21 mV in V_1/2ACT_ (n = 13) compared to BK:LINGO1 or BK:LINGO2 currents (Figs. 4*F* and 5), this was not significantly different to the other chimeras with a LINGO1 ITD. The V_1/2ACT_ of BK:LINGO chimeras with a LINGO2 ITD (BK:122, 97 ± 14 mV, n = 7; BK:112, 91 ± 17 mV, n = 6; BK:212, 112 ± 18 mV, n = 11) were similar to BK:LINGO2 currents (125 ± 12 mV, n = 9, Figs. 4, *C*, *E*, and *H* and 5) in 100 nM Ca^2+^.

The V_1/2ACT_ in 1 μM Ca^2+^ of all chimeras with a LINGO1 ITD (BK:211, 76 ± 12 mV, n = 12; BK:221, 78 ± 13 mV, n = 13; BK:121, 70 ± 12 mV, n = 9) were similar to BK:LINGO1 in 1 μM Ca^2+^ (78 ± 14 mV, n = 11, *p* ≥ 0.81; Figures 4, *A*, *C*, and *E* and 5). In comparison, the V_1/2ACT_ for BK:LINGO2 (53 ± 19 mV, n = 9, Figs. 4*B* and 5) was left-shifted compared to BK:LINGO1. All chimeras with a LINGO2 ITD had a left-shifted V_1/2ACT_ similar to BK:LINGO2 (BK:122, 39 ± 9 mV, n = 7; BK:112, 40 ± 10 mV, n = 6; BK:212, 57 ± 24 mV, n = 11; *p* ≥ 0.34) in 1 μM Ca^2+^. Taken together, these data suggest that in 1 μM Ca^2+^, the ITD appears to be the major determinant of V_1/2ACT_ induced by coexpression of BKα with LINGO1 and LINGO2, respectively.

Consistent with this idea, swapping ED or TMD in 1 μM Ca^2+^ had very little effect on V_1/2ACT_. For example, the V_1/2ACT_ for BK:LINGO1 (78 ± 14 mV, n = 11) was similar to BK:121 (70 ± 12 mV, n = 9), whereas BK:LINGO2 (53 ± 19 mV, n = 9) was like BK:212 (57 ± 24 mV, n = 11). Similarly, for the ED swaps, the V_1/2ACT_ for BK:122 (39 ± 9 mV, n = 7) and BK:211 (76 ± 12 mV, n = 12) were not different to the WT BK:LINGO2 and BK:LINGO1 currents, respectively in 1 μM Ca^2+^ (Figs. 4 and 5).

In LINGO1, the last 8 C terminus residues of the ITD are essential for inactivation, but do not contribute significantly to the V_1/2ACT_ (9, 10). We therefore wished to test if the same held true for the chimeras, since these are likely to be useful constructs to further study BK LINGO interactions. For these experiments, we used the LINGO211 chimera as it has several advantages. For example, in only 5% of patches are BK:LINGO1 currents greater than 1 nA (10), making it difficult to generate robust GV curve data. Conversely, although BK:LINGO2 yields abundant currents, they can readily oxidize, resulting in a rapid loss of inactivation (9). With the BK:211 chimera, neither of these issues are prevalent and we were able to record robust currents to study the interaction of LINGO with BK channels. Deleting the distal 8 amino acids (RKFNMKMI) of BK:211 (BK:211_ΔRKFNMKMI_) abolished inactivation and the V_1/2ACT_ was 113 ± 13 mV in 100 nM Ca^2+^ (n = 8), which was slightly left-shifted compared to BK:211 (124 ± 10 mV, n = 12) and BK:LINGO2 (125 ± 12 mV, n = 9) under identical recording conditions. This suggests that the negative shift in activation was retained in the absence of the last eight residues and is conferred by residues proximal to the inactivating domain in the ITD. This is consistent with previous studies on BK:LINGO1 which showed that the negative shift in inactivation was retained with deletion of residues up to and including 600 to 620 in the distal C terminus (10). In contrast to the effect of the ITD on V_1/2ACT_, we observed little consistent effect of any domain swap on the voltage for half maximal steady-state inactivation (V_1/2INACT_, Fig. S10).

## Discussion

We used a chimeric approach to define the contribution of the ED, TMD, or ITD in the control of BK channel cell surface expression and V_1/2ACT_. Our data reveal that the ED of LINGO1 is critical for suppression of BKα trafficking to the plasma membrane, but this effect is independent of the LRR in the ED. In contrast, the ITD is an important determinant of the shift in V_1/2ACT_ of BK:LINGO currents between BK:LINGO1 and BK:LINGO2 at different Ca^2+^ and compared to BKα alone.

### ED of LINGO1 controls BK channel surface expression

The data showed that the ED of LINGO1, but not LINGO2 determined the cell surface expression of BKα-subunits. As LINGO1 does not affect BKα internalization, this would suggest the major effect of BK assembly with LINGO is to retard forward trafficking, or recycling, of the BK:LINGO1 complex. This raises a number of fundamental questions such as where in the cell surface trafficking pathway do BK channels assemble with LINGO1, or LINGO2, and at which stage(s) does LINGO1-mediated inhibition of surface expression occur (*i.e.* ER, Golgi, and or transport vesicles)? Furthermore, how does the presence of the lumenal ED of LINGO1, but not LINGO2, control plasmalemmal expression of BKα? Deletion of the 12 LRRs alone in the LINGO1 ED did not prevent suppression of BKα surface expression in the LINGO1 deletion mutant ΔLRRC. These data suggest that a combination of multiple subdomains of the LINGO1 ED is important for the inhibitory effect of LINGO1 on BK channel surface expression. In support of this model, subdomain swaps of the LINGO2-CT2 or LINGO2-Ig2 subdomains in LINGO1-ΔLRRC abrogated the inhibitory effect on BK channel surface expression, whereas swap of either the -CT2 or Ig2 subdomains in full-length LINGO1 did not disrupt the suppression of BK channel surface expression. It is interesting to note that these regions are the least conserved between LINGO1 and LINGO2 (Fig. S1 and (14, 15)).

The structure of the isolated LINGO1 ED, without the TMD and ITD, has been solved (13) revealing a tetrameric assembly. In this structure, each ED is aligned head to tail to form a ring that would be predicted to lie parallel with the plasma membrane with the IG-like domain at right angles to the membrane. This is reminiscent of the recent structural insights gained from BK:γ1 complexes which also adopt a tetrameric ring of head to toe “dominos” (17, 18, 19). These data suggest very little contact between the ED and BKα-subunits, although interaction of the LRR-CT domain and N terminus of BK may occur (17). It is unclear if the ED of LINGO proteins will adopt a similar structure when assembled with BK, given that they contain 6 more LRR domains than the γ-subunits. The main interaction of γ1-subunits with BK appears to involve the TMD, an extracellular “hook” and intracellular polybasic region at either end of the γ1 transmembrane (TM) helix (17, 18, 19).

Since deletion of the LRRC domain in the LINGO1 ED still suppressed BKα plasmalemmal expression and produced inactivating currents, this would suggest that the normal tetrameric arrangement of the isolated LINGO1 ED *per se* is not important. However, since other regions in the LINGO1 ED were required for suppression, coupled with the fact that domain swaps of the TMD or ITD had no effect on BKα trafficking, suggests that the assembly of the LINGO1 ED as a tetramer is not essential. Whether a 4:4 ratio of BK:LINGO1 subunits is required for the downregulation of plasmalemmal expression, or if it can be suppressed by a single LINGO1 subunit in the BKα tetramer complex remains to be determined. However, we cannot exclude that potential assembly of LINGO1 early in the biosynthetic pathway might disrupt assembly of BK channels contributing to suppressed delivery of functional BK channels to the cell surface. Elucidation of how and where LINGO regulatory subunits assemble with BK channels in the biosynthetic/trafficking pathways to the plasma membrane is required.

In this context, the inhibition of cell surface expression of the ZERO variant of BKα by LINGO1 is in contrast to the enhanced surface expression of this variant promoted by assembly with either β1- or β4-subunits (16, 20, 21). Since both β1 and β4 both promote exit of BKα from the endoplasmic reticulum (ER), it appears logical that LINGO1 could retard BKα exit from the ER. Moreover, as γ and LINGO1 subunits coassemble with BK channels that also contain β-subunits (10, 17, 18), it is possible that complex interactions in the control of BK channel surface expression and biophysical properties are dependent on cell-specific differential multimeric assembly of these subunits.

How the lumenal ED domain of LINGO1, but not LINGO2, may retard forward trafficking of BK remains to be determined. It appears unlikely that the ED of LINGO1 would interact with typical coat proteins required for anterograde trafficking. However, it is important to highlight that for transmembrane proteins, multiple mechanisms control both ER and Golgi trafficking, including the partitioning of TMDs of transmembrane proteins into lipid bilayers of different composition to allow TMDs to sense bilayer thickness/order (22, 23, 24, 25). Indeed, both the composition of ER exit sites as well as partitioning of TMDs in ordered lipid domains of Golgi membranes have been reported to control trafficking of transmembrane proteins (23, 24, 25). It is thus tempting to speculate that, upon assembly of LINGO1 and BK channel subunits, the ED of LINGO1 controls the orientation of the LINGO1 and/or BK channel TMDs, allowing the complex to partition into different lipid bilayer environments to control trafficking.

### ITD controls V_1/2ACT_ in LINGO subunits

Our chimeric approach also revealed that the ITD of LINGO1 and LINGO2 is the region most likely to determine the negative shift in the V_1/2ACT_ of BK:LINGO currents compared to BKα alone and the difference between LINGO1 and LINGO2 seen at different Ca^2+^. Importantly the difference in V_1/2ACT_ between BK:LINGO1 and BK:LINGO2 currents was essentially retained by swapping the respective ITD in the chimeras even though the effect of LINGO1 and LINGO2 *per se* are different at 100 nM and 1 μM Ca^2+^. Since removal of the last 8 amino acids of the ITD abolished inactivation but shifted V_1/2ACT_ more negatively, albeit modestly, this suggests that the relatively short sequence between the end of the TMD domain and the inactivation octet is a critical determinant in the difference of V_1/2ACT_ between LINGO1 and LINGO2. Furthermore, Dudem *et al.*, (10) demonstrated that the negative shift in V_1/2ACT_ was also retained when residues 600 to 620 were deleted from the LINGO1 tail. Intriguingly, recent structural insights of γ1:BK complexes and previous biophysical analysis of γ-subunit chimeras (17, 18, 19, 26) revealed that a polybasic stretch of arginines, close to the intracellular membrane was an important determinant of V_1/2ACT_. Although LINGO1 and LINGO2 contain three (**R**G**K**GNT**K**) and four (**R**G**K**G**K**H**K**) positively charged residues in the juxtamembrane region, respectively, sequence alignment with the γ-subunits (10) suggest that only the last three of these residues are likely to be at the start of the ITD in LINGO1 and LINGO2. Interestingly, a comparison of different γ-subunits reveals that the degree of shift in V_1/2ACT_ correlates with the number of basic residues in the polybasic stretch ITD juxtaposed the TMD (17, 26). If a similar mechanism were present in LINGO proteins, one might expect that the V_1/2ACT_ of LINGO2 would be more negative than LINGO1, which is not the case (9, 10). However, until the high-resolution structures of the TMD and ITD of LINGO proteins are obtained, the precise interactions of the residues involved in setting the V_1/2ACT_ remains speculative. Recent structural insights of γ-subunit interaction with BKα suggest at least three potential models for the juxtamembrane region to control V_1/2ACT_. First, Redhardt *et al.*, (18) proposed, from cryo-EM analysis of rabbit BK:γ1 complexes, that the polybasic region lies close to the voltage-sensing domain of BK and the presence of these immobile positive charges, thus might act to repulse the gating charges, facilitating transition of the voltage-sensing domain (VSD) to the active conformation. This would be in agreement with the lack of effect of γ-subunits on Ca^2+^ sensitivity in BK:γ1 complexes and the shift resulting from increase in the allosteric coupling of VSD activation and pore domain opening (26). Second, although it was based on a lower resolution structure, Yamanouchi *et al.*, (19) suggested that the polybasic region interacts directly with the Ca^2+^-sensing RCK domains. Whether the interaction is direct or not, it is clear that mutation of key residues in RCK (D370) and γ1 (R295) did reduce the effect of γ1 on V_1/2ACT_. Finally, polybasic regions provide a mechanism to alter the orientation of the juxtaposed TMD in relation to the lipid bilayer through interaction with negatively charged phospholipids (22). It will be interesting to examine whether mutation of any of the charged residues in the juxtamembrane region of LINGO proteins control the difference in the V_1/2ACT_ observed between BK:LINGO1 and BK:LINGO2 currents. Furthermore, as BK channels can be regulated by negatively charged phospholipids, such as PIP_2_, whether the polybasic stretch in LINGO1 confers sensitivity to PIP_2_ is an intriguing possibility and remains to be explored. It is also interesting to speculate that if the LINGO proteins adopt a similar and extended intracellular helix to γ1 (19), then the conserved, positively charged lysine residue in LINGO1-4 (at the equivalent position of R295 in γ1) could orientate and interact with the same region of the BK channel. However, it seems unlikely that the presence of a positively charged lysine in this position in LINGO1-4 alone could explain the differences observed in V_1/2ACT_ in BK:LINGO proteins.

Although swap of the TMD between LINGO1 and LINGO2 did not significantly modify the V_1/2ACT_ of BK:LINGO1 compared to BK:LINGO2 currents, the TMD may still play a role in the negative shift of V_1/2ACT_ compared to BKα. In this regard, Li *et al.* (26) used a chimeric approach with γ1-4 to reveal that a phenylalanine (F273) in the TMD of the γ1 accessory subunit also controlled the negative shift in V_1/2ACT_ induced by coassembly of γ-subunits with BKα. Again recent structural insight of γ1:BKα complexes reveal the importance of the kinked shape of the γ1 TM to allow it to interface across the activated VSD of BKα, with F273 in γ1 being a key interaction point (17, 18, 19). A phenylalanine is present in the equivalent location in LINGO1 and is replaced by a cysteine residue in LINGO2 (Fig. S1). Interestingly, a phenylalanine is immediately downstream of this cysteine in the linear LINGO2 sequence. In γ1-subunits, mutation of F273 also significantly reduces assembly of γ1 with BK (17), potentially resulting in complexes with a stoichiometry of <4. Although LINGO1 and LINGO2 lack the proline in γ1 upstream of the phenylalanine that contributes to the kink in the γ1 TMD, they both have a glycine residue at this position which could cause a similar structural change to the proline in γ1. Thus, future studies should address whether the equivalent residue to the γ1 F273 in the TM of LINGO is also important in determining V_1/2ACT_, complex assembly and the orientation of the LINGO TM in the BK complex. Finally, in agreement with chimera studies in γ-subunits (26), the ED of LINGO1 or LINGO2 appear to have no significant impact on V_1/2ACT._ Taken together these data suggest that the juxtamembrane region of the ITD in both LINGO and γ-subunits may contribute to the V_1/2ACT_ of BK channels complexed with both γ-subunits and LINGO subunits, but conserved residues in the TMD domain may also contribute to this.

Future work to delineate how LINGO1 controls both the biophysical properties and trafficking of BK channels will require an integrated electrophysiological, cryo-EM structural biology, imaging, and biochemical approach. This should allow us to understand the spatial organization of LINGO1 interrogate the molecular interactions between LINGO1 and BK channel subunits to control V_1/2ACT._

### Physiological relevance of LINGO1-mediated regulation of BK channels

BK channels are ubiquitously expressed in the majority of cell types in the body (1, 2, 3, 4, 5, 6, 7), whereas LINGO proteins have more discrete expression patterns predominantly in the nervous system (14, 15, 27, 28, 29). Nevertheless, increasing evidence suggests that some LINGO isoforms may also be present in a variety of endocrine, epithelial, smooth muscle, and pacemaker cells (30, 31, 32, 33). This likely provides another mechanism to diversify the function and properties of BK channels by coassembly with LINGO1 in a tissue and cell type–specific manner. As LINGO1 confers both inactivation and suppression of cell surface expression it likely acts as a “functional inhibitor,” of BK channel physiology in a number of systems (10). Indeed, in the brain overexpression of LINGO1 has been implicated in both essential tremor as well as movement disorders such as Parkinson’s disease (10, 34, 35, 36, 37, 38, 39, 40, 41). Pharmacological inhibition (42, 43) or genetic ablation of BK channels (44, 45, 46) also results in tremor and motor dysfunction.

In summary, using the distinct characteristics of LINGO1 and LINGO2 on BK channel properties, we have used a chimera approach to reveal the critical role of the LINGO1 ED in controlling plasmalemmal expression of BKα and that the ITD of LINGO1 and LINGO2 are critical determinants of the V_1/2ACT_ of LINGO:BK currents.

## Experimental procedures

### Electrophysiology

#### Solutions

All excised patch experiments were performed at 37 ^o^C in 140 mM symmetrical K^+^ solutions which contained 140 mM KCl, 10 mM glucose, 10 mM Hepes, and either 1 mM EGTA (for free [Ca^2+^] 100 nM to 300 nM) or 1 mM HEDTA (for free [Ca^2+^] 1 μM to 10 μM), and the pH of all solutions was adjusted to 7.2 with KOH. These solutions were made up in double distilled, deionized, filtered water from a MilliQ water purification system. The pipette solution contained 100 nM free Ca^2+^. We used “chelator” to calculate the total amount of Ca^2+^ required, as per Schoenmakers *et al.* (47) and free Ca^2+^ concentrations were checked with a Ca^2+^-sensitive electrode.

During experiments, the dish containing HEK cells was superfused with Hanks solution which contained (in mM) 129.8 Na^+^, 5.8 K^+^, 135 Cl^-^, 4.17 HCO_3_^-^, 0.34 HPO_4_^2-^, 0.44 H_2_PO_4_^-^, 1.8 Ca^2+^, 0.9 Mg^2+^, 0.4 SO_4_^2-^, 10 glucose, 2.9 sucrose and 10 Hepes, and its pH was adjusted to 7.4 with NaOH. In addition, the patch under study was continuously superfused by means of a close delivery system consisting of a pipette (tip diameter 200 μm) placed approximately 300 μm away from the cell. This could be switched, with a dead-space time of around 5 s, to a solution containing a drug.

#### Patch clamp electrophysiology

Electrodes were pulled from Corning borosilicate glass (1.5 mm O.D. × 0.86 mm I.D.) using a Sutter P-97 pipette puller and fire polished using a Narashige MF 83 Microforge. Pipettes had resistances of 2 to 5 MΩ when filled with recording solutions and series resistance was compensated by up to 80%. Standard single-channel patch clamp recording methods were used. Voltage clamp commands were delivered *via* an Axopatch 200B patch clamp amplifier (Axon Instruments) connected to a Digidata 1322A AD/DA converter (Axon Instruments) interfaced to computers running pClamp software (Axon Instruments, https://www.moleculardevices.com/products/axon-patch-clamp-system/acquisition-and-analysis-software/pclamp-software-suite). Data were acquired at 100 kHz and filtered at 2 kHz or 5 kHz. Patches were held at either −60 mV or −100 mV and depolarized in 20 mV increments to 200 mV. Residual capacitance and leakage currents were subtracted using either a P/4 protocol or offline by manual leak subtraction.

For the majority of electrophysiological recordings, the α-subunit of the rabbit BK channel (rBK) was used as described previously (9, 10), which corresponds to the ZERO variant of mouse BKα and to variant 2 (NM_002247.3) of human BKα. We used 100:150 ng ml^-1^ cDNA for BKα:GFP only transfections, whereas 100:500:150 ng ml^−1^ cDNA ratio was used for BK:LINGO2:GFP. For BK:LINGO1:GFP and BK:chimera:GFP, cotransfections the cells were transfected in a 300:300:150 ng ratio.

cDNA was transiently transfected into HEK293 cells using lipofectamine. HEK293 cells were cultured in Dulbecco's modified Eagle's medium + minimal essential medium containing 10% foetal bovine serum and 1% penicillin, streptomycin antibiotics at 37 ^o^C and 95% humidifying incubator containing 5% CO_2_. Subculturing was done by 0.05% trypsin-EDTA solution.

#### Electrophysiology curve fitting and statistics

Conductance (G) was derived from peak currents according to Ohm's law

(1) $$G=\frac{I}{\left(V-E_{K}\right)}$$

where *E*_K_ = 0 mV in symmetrical [K^+^]. *G–V* relationships were fitted with the Boltzmann equation

(2) $$\frac{G}{G_{\mathrm{MAX}}}=\frac{1}{1+e\left(\left(Vm-V_{1/2}\right)/S\right)}$$

where *V*_1/2_ is the voltage of half-maximum activation, *S* is the slope of the curve, V_*m*_ the test potential, G the conductance, and G_*max*_ the maximal conductance. For BK constructs, data from each patch was normalized to the peak conductance measured in either 1 μM or 10 μM Ca^2+^ to obtain G_max_ and curves were constrained to the G_*max*_ value obtained in this. In full-length BKα:LINGO2 channels, currents were normalized to the peak G_*max*_ recorded in 1 μM Ca^2+^ or 100 nM Ca^2+^.

Inactivation curves were fitted with a similar Boltzmann function:

(3) $$I/I_{max}=1/\left\{1+\exp\left[\pm \left(V_{1/2}-V_{c}\right)/K\right]\right\}$$

where *I* was the current recorded at the test step, *I*_max_ was the maximal current recorded, *V*_c_ was the conditioning potential, and *K* was the slope factor.

### Generation of LINGO chimeras

The HA epitope sequence (YPYDVPDYA) was engineered into the ED of full-length LINGO1 in PCMV6 and LINGO2 in pcDNA3 clones by PCR, at amino acid position 42 in LINGO1 and amino acid position 28 in LINGO2, downstream of the signaling sequence using the following primers as described previously (9):

LINGO1-HA-fwd: TAC CCA TAC GAT GTT CCA GAT TAC GCT tgcccgccccgct

LINGO1-HA-rev: AGCGTAATCTGGAACATCGTATGGGTAgcccgtggccga.

LINGO2-HA-fwd: TAC CCA TAC GAT GTT CCA GAT TAC GCTtgccccgctcgct.

LINGO2-HA-rev: AGCGTAATCTGGAACATCGTATGGGTAgccaatggtgga.

HA-tagged LINGO chimera clones were created by synthesizing fragments and subcloning into LINGO1-HA or LINGO2-HA full-length expression plasmids or by direct synthesis and cloning into expression vectors. Restriction enzyme recognition sequences were included in the synthesized fragments to allow subcloning into full-length backbones. All chimeras were fully sequenced on both strands to confirm sequence.

#### Generation of ED, TMD, and ITD swap chimeras: LINGO112, LINGO122, LINGO121, LINGO221, LINGO212, and LINGO211

To generate chimeras with the ED of LINGO1 with different TMD and ITD LINGO1-HA pCMV6 was digested with BamHI and XhoI generating two fragments 1927 bp and 4829 bp. To generate LINGO112 and LINGO222, the 1927 bp fragment was further digested with NcoI generating fragments 179 bp and 1748 bp. A denovo synthesized fragment spanning LINGO1 TMD and LINGO2 ITD (L1-TMD_L2-ITD) and LINGO2 TMD and LINGO2 ITD (L2-TMD_L2ITD) were synthesized by Twist Bioscience with Nco1 and Xho1 restrictions sites were digested with NcoI and XhoI and were ligated to the 1748 bp fragment and the 4829 bp plasmid fragment to generate LINGO112 and LINGO122, respectively. To generate LINGO121 a full-length HA-tagged LINGO221, chimera was synthesized by Twist Bioscience with an engineered Nco1 site at the start of the TMD without changing sense, digested with NcoI and XhoI, and the fragment spanning the TMD of LINGO2 TMD and LINGO1 ITD was subcloned into LINGO1-HA pCMV6. Site-directed mutagenesis was used to reverse the single amino acid changed in the L2TM sequence caused by utilizing the NcoI site at the beginning of the TM domain.

To generate chimeras with the ED of LINGO2 and different TMD and ITD, LINGO212 was created by subcloning fragment L1-TM_L2-ID into the LINGO221 backbone using NcoI and XhoI. To create LINGO211, the fragment L1TM_L1IN was subcloned from LINGO1-HA using NcoI and XhoI and ligated into LINGO2-HA.

#### Generation of LINGO1 and LINGO2 ED subdomain chimeras

LINGO1-ΔLRR lacking the LRRs (residues 72–368 deleted) was generated as previously described (9), and the HA tag inserted at position 42 as for LINGO1 by PCR. LINGO1 with engineered LINGO2 subdomains in the ED were generated as follows: LINGO1-L2Nter: The N-terminal fragment of LINGO2 synthesized by GenScript with restriction sites KpnI/PpuMI and was subcloned into LINGO1-HA. Full-length LINGO1_L2CT was synthesized by GenScript in pcDNA3.1 and LINGO1-stalk2 by Twist Bioscience in pTwist-CMV. LINGO1_L2Ig was generated using a fragment synthesized by GenScript with flanking PmlI/NotI restriction sites and subcloned into LINGO1-HA. LINGO2 with engineered LINGO1 subdomains in the ED were generated as follows: LINGO2-L1Nter: the N-terminal fragment of LINGO1 synthesized by GenScript with restriction sites KpnI/EcoRI was subcloned into LINGO2-HA. Full-length LINGO2_L1CT was synthesized by GenScript in pcDNA3.1 and LINGO2-stalk1 by Twist Bioscience in pTwist-CMV. LINGO2_L1Ig was generated using a fragment synthesized by GenScript with flanking EcoNI/PpuMI restriction sites and subcloned into LINGO2-HA.

### BK channel cell surface and internalization assays

#### HEK 293 cell culture and OCW assays

HEK 293 cells were maintained in Dulbecco's modified Eagle's medium containing 10% foetal bovine serum (GM) and incubated at 37 °C in 5% CO2. Cells were transfected with using Polyjet (Tebubio) 24 h after plating in 6-well plates with the ZERO, or STREX, variant of the BK channel α-subunit with an extracellular (N terminal) Flag-epitope tag and a CT (intracellular) -myc epitope tag (9). Coexpression with HA-tagged LINGO1, LINGO2, or respective chimera OCW were conducted at a ratio of 0.5 μg the respective LINGO construct (or empty vector) to 1 μg ZERO or STREX unless otherwise indicated. OCW assays were conducted essentially as previously described (10, 20) but using Flag-BK-myc–tagged channels in quadruplicate in each independent assay. Twenty-four hours after transfection, cells were replated in 96-well plates (Greiner, clear-bottomed, black-sided wells, poly-d-lysine–coated) and 24 h later were stained on ice with mouse anti-FLAG-M2 (Sigma, 1:100 in GM) for 2 h, washed and incubated with IRDye800CW Goat Anti-Mouse IgG (Licor, 1:100 in GM) for 1 h on ice. All steps from this point were carried out in the dark. After washing, cells were fixed in 3.7% formaldehyde solution in PBS for 20 min, washed, and permeabilized with PBS containing 0.1% (v/v) Triton X-100 before blocking in Odyssey blocking buffer (OBB; Licor) for 1 h or overnight. Anti-myc antibody (ICL Inc. 1/5000 in OBB) was applied for 1 h at room temperature, washed with PBS + 0.1% Tw and anti-Rabbit secondary IRDye680RD (Licor, 1:1000 in OBB) applied for 1 h at room temperature. To determine cell number, TO-PROTM-3-Iodide (1:500) was applied to separate wells. Cells were washed in PBS+0.1% Tween before imaging on an Odyssey M Imager with focus offset set to 3.8 mm. Images were analyzed using Image Studio Lite Ver5.2 (Licor, https://www.licorbio.com/image-studio-lite). Staining intensity was normalized to cell number and background subtracted before calculating the ratio of surface Flag:myc total protein staining.

#### Internalization assay

Transfected HEK293 cells were plated in 96-well plates (Greiner, clear-bottomed, black-sided wells, poly-d-lysine–coated) as for OCW assays and internalization assays performed essentially as previously described (20). Twenty-four hours after replating, all wells were stained on ice with anti-FLAG-M2 (Sigma, 1:100) for 2 h. The cells were then washed and incubated with IRDye800CW goat anti-mouse IgG (Licor, 1:1000 in GM) for 1 h on ice and protected from light. The cells were then washed, and wells for determination of internalization at time 0 were exposed to ice-cold stripping buffer (0.1 m glycine, 0.1 m NaCl, pH 2.5, in PBS) for 15 min and then washed in growth medium. To determine internalization, labeled and unstripped cells were incubated at 37 °C for 1 h. The cells were then cooled on ice for 5 min before applying ice-cold stripping buffer for 15 min to wells measuring internalization, whereas a subset remained unstripped to measure total channel. All wells were then stained with NucRed Live 647 ReadyProbes reagent (Thermo Fisher Scientific) for 30 min on ice. Cells were washed then imaged on an Odyssey M Imager, focus offset at 3 mm, intensity 5.0 for both 700 and 800 channels. Images were analyzed using Image Studio Lite version 5.2 (freeware from Licor). For each well, staining intensity in the 800 channel was normalized to the NucRed 700 channel signal obtained for each well. Background signal detected in time 0 stripped cells (*i.e.,* no internalization) was averaged and subtracted from the other wells. Internalization after 60 min was then normalized to the total surface expression at time 0 to express internalization as a percentage of total BK channel surface expression before internalization.

##### Statistical tests

All data are presented as mean ± SD and was analyzed with Prism software (GraphPad, www.graphpad.com). Individual data points are also shown in bar charts. Statistical differences were assessed using either paired or unpaired *t* tests (Wilcoxon signed-rank test, Mann–Whitney test), as appropriate. ANOVA was used for three or more parametric data sets with Dunnett’s or Tukey tests where appropriate. Comparisons of three or more nonparametric data sets used Friedman’s test, followed by Dunn’s test for multiple comparisons or Kruskal–Wallis with *post hoc* Dunn’s test where appropriate. *p* < 0.05 was taken as significant.

## Data availability

All data are available upon request to M. J. Shipston at mike.shipston@ed.ac.uk or M. A. Hollywood at mark.hollywood@dkit.ie.

## Supporting information

This article contains supporting information (Figs. S1–S10).

## Conflict of interest

The authors declare that they have no conflicts of interest with the contents of this article.
