# Supplementary material for: Distinct domains of LINGO1 control surface expression and biophysical properties of large conductance Ca2+- and voltage-activated potassium (BK) channels
Source: J Biol Chem. 2025 Aug 5;301(9):110550. doi: 10.1016/j.jbc.2025.110550 (PMC12423400; doi:10.1016/j.jbc.2025.110550)
Supplement: Supplementary Figures [file mmc1.pdf]

Figure S1

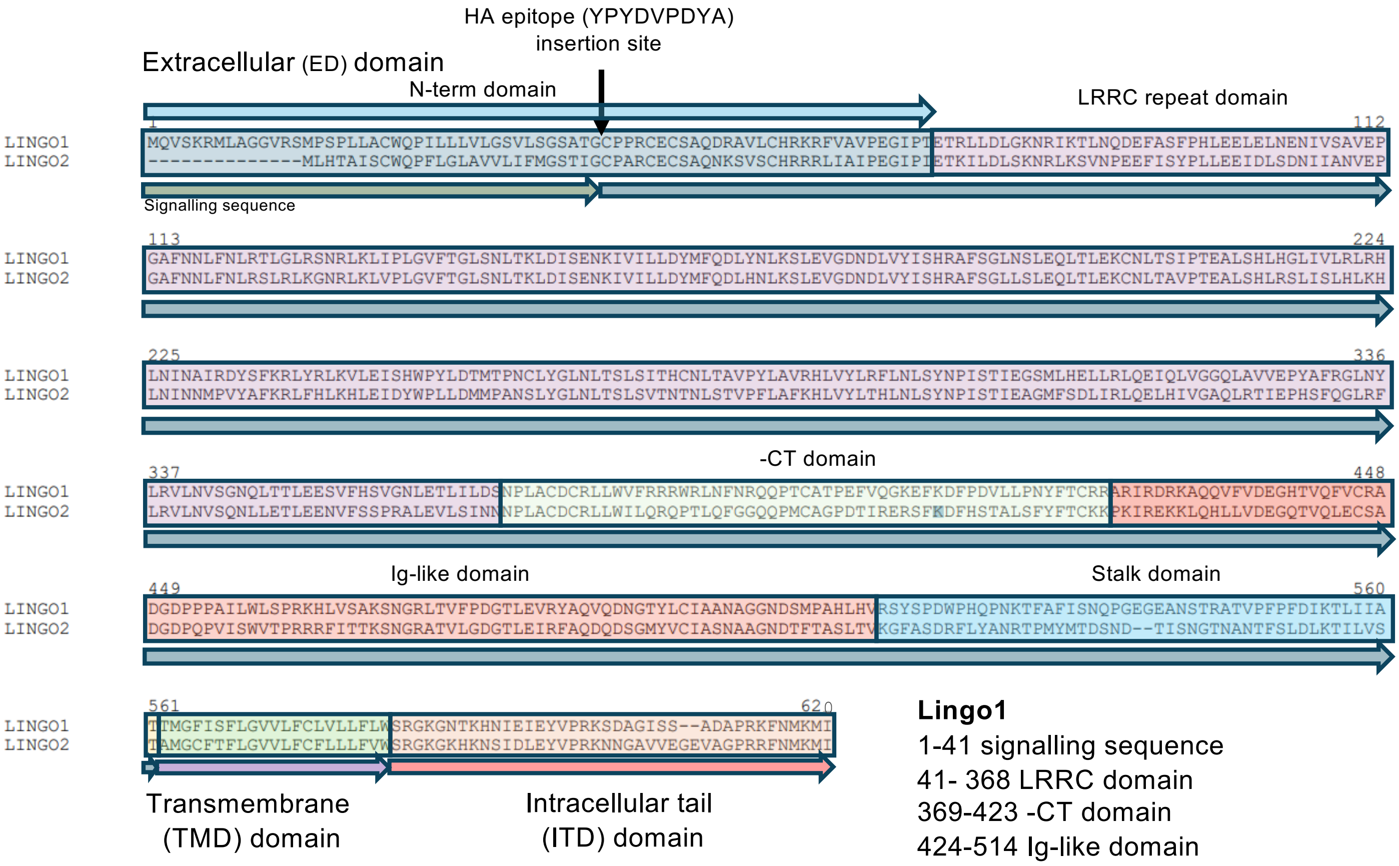

Lingo1
1-41 signalling sequence
41- 368 LRRC domain
369-423 -CT domain
424-514 Ig-like domain
515-561 stalk domain
562-582 transmembrane (TMD) domain
583-620 intracellular (ITD) domain

Figure S2

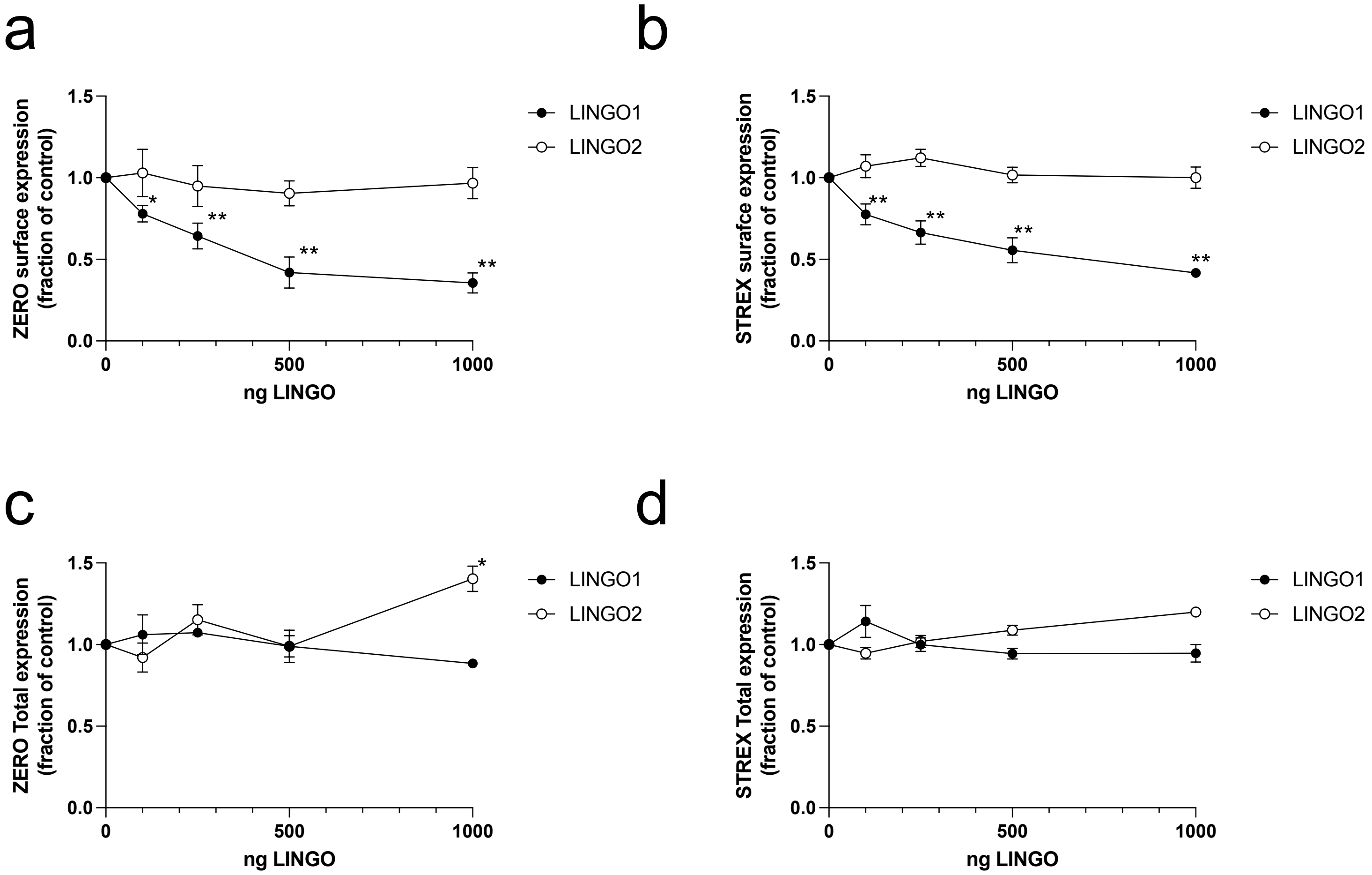
a
ZERO surface expression (fraction of control)
ng LINGO
LINGO1
LINGO2
b
STREX surafce expression (fraction of control)
ng LINGO
LINGO1
LINGO2
c
ZERO Total expression (fraction of control)
ng LINGO
LINGO1
LINGO2
d
STREX Total expression (fraction of control)
ng LINGO
LINGO1
LINGO2

Figure S3

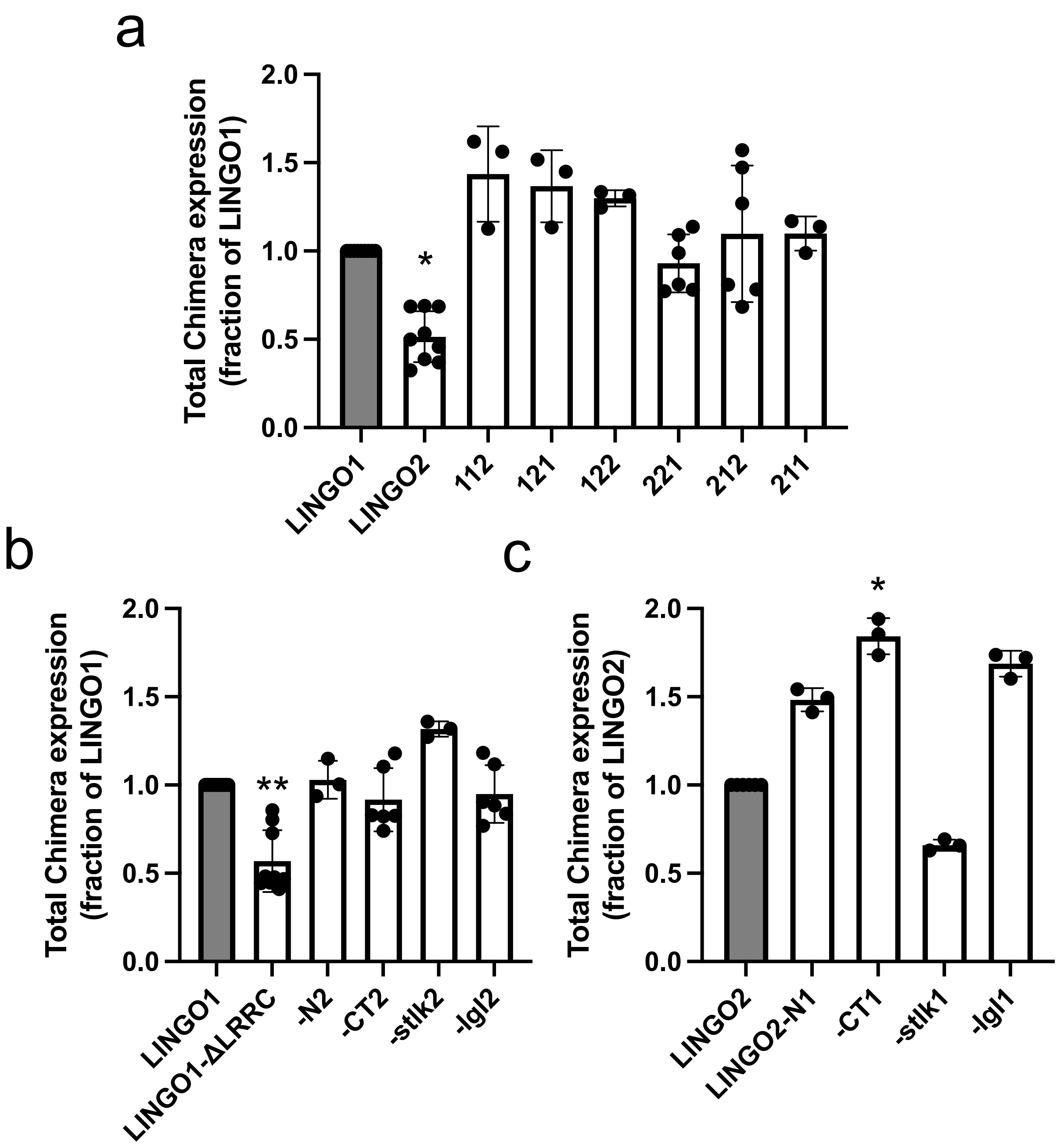
a
Total Chimera expression (fraction of LINGO1)
2.0
1.5
1.0
0.5
0.0
*
LINGO1
LINGO2
112
121
122
221
212
211
b
Total Chimera expression (fraction of LINGO1)
2.0
1.5
1.0
0.5
0.0
**
LINGO1
LINGO1-ΔLRRC
-N2
-CT2
-stlk2
-Igl2
c
Total Chimera expression (fraction of LINGO2)
2.0
1.5
1.0
0.5
0.0
*
LINGO2
LINGO2-N1
-CT1
-stlk1
-Igl1

Figure S4

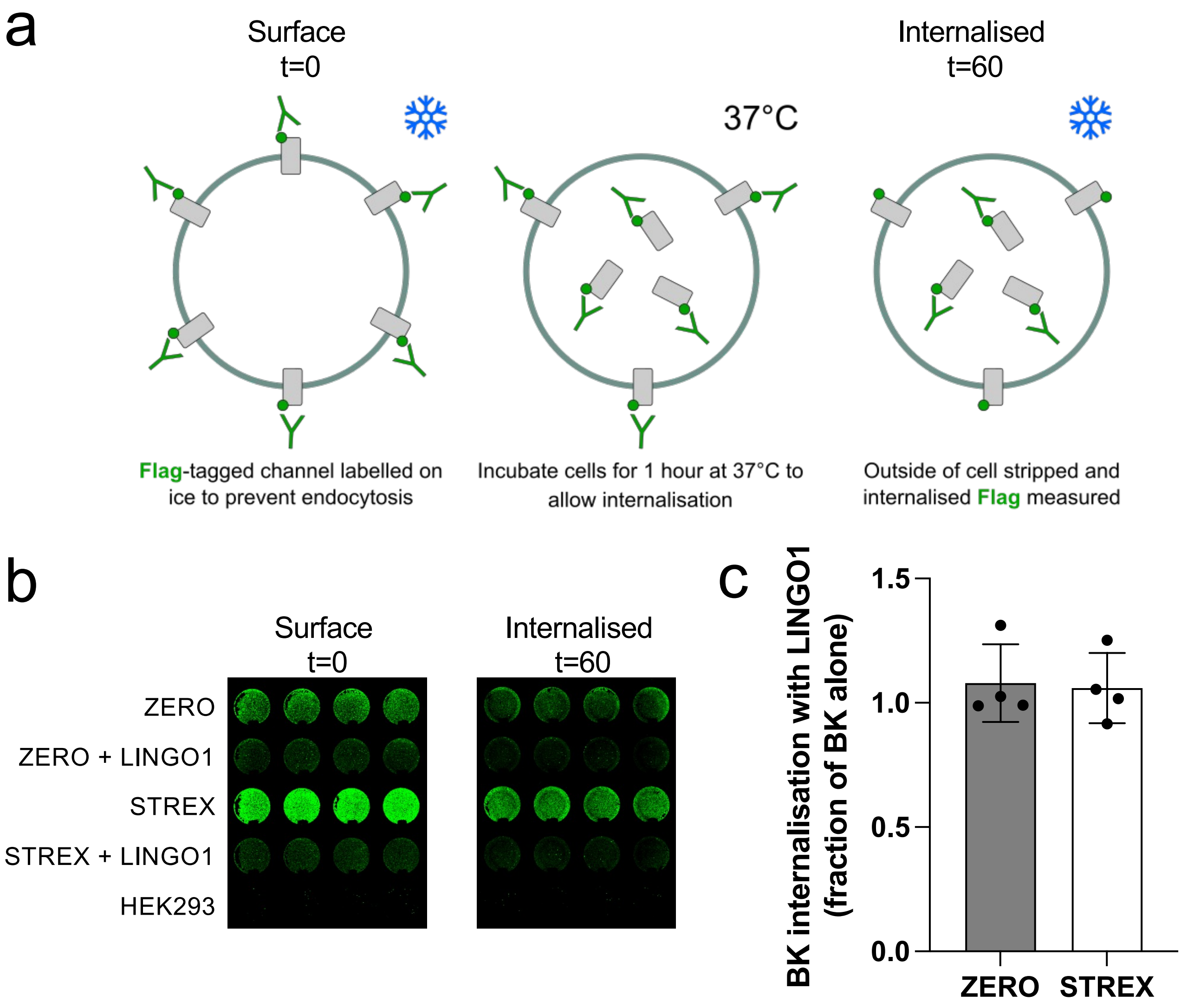
a
Surface
t=0
37°C
Internalised
t=60
Flag-tagged channel labelled on ice to prevent endocytosis
Incubate cells for 1 hour at 37°C to allow internalisation
Outside of cell stripped and internalised Flag measured
b
Surface
t=0
Internalised
t=60
ZERO
ZERO + LINGO1
STREX
STREX + LINGO1
HEK293
c
BK internalisation with LINGO1
(fraction of BK alone)
1.5
1.0
0.5
0.0
ZERO
STREX

Figure S5

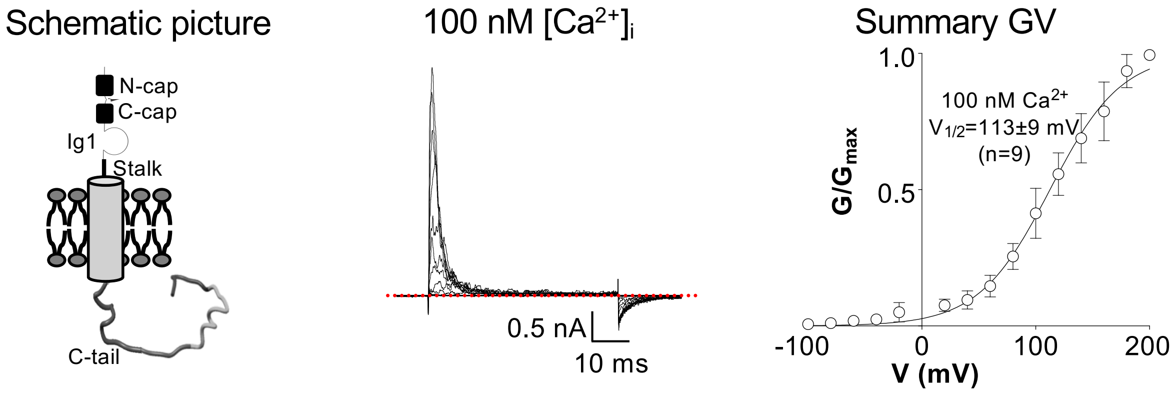
Schematic picture
N-cap
C-cap
Ig1
Stalk
C-tail
100 nM [Ca2+]i
0.5 nA
10 ms
Summary GV
1.0
0.5
G/Gmax
100 nM Ca2+
V1/2=113±9 mV
(n=9)
-100
0
100
200
V (mV)

Figure S6

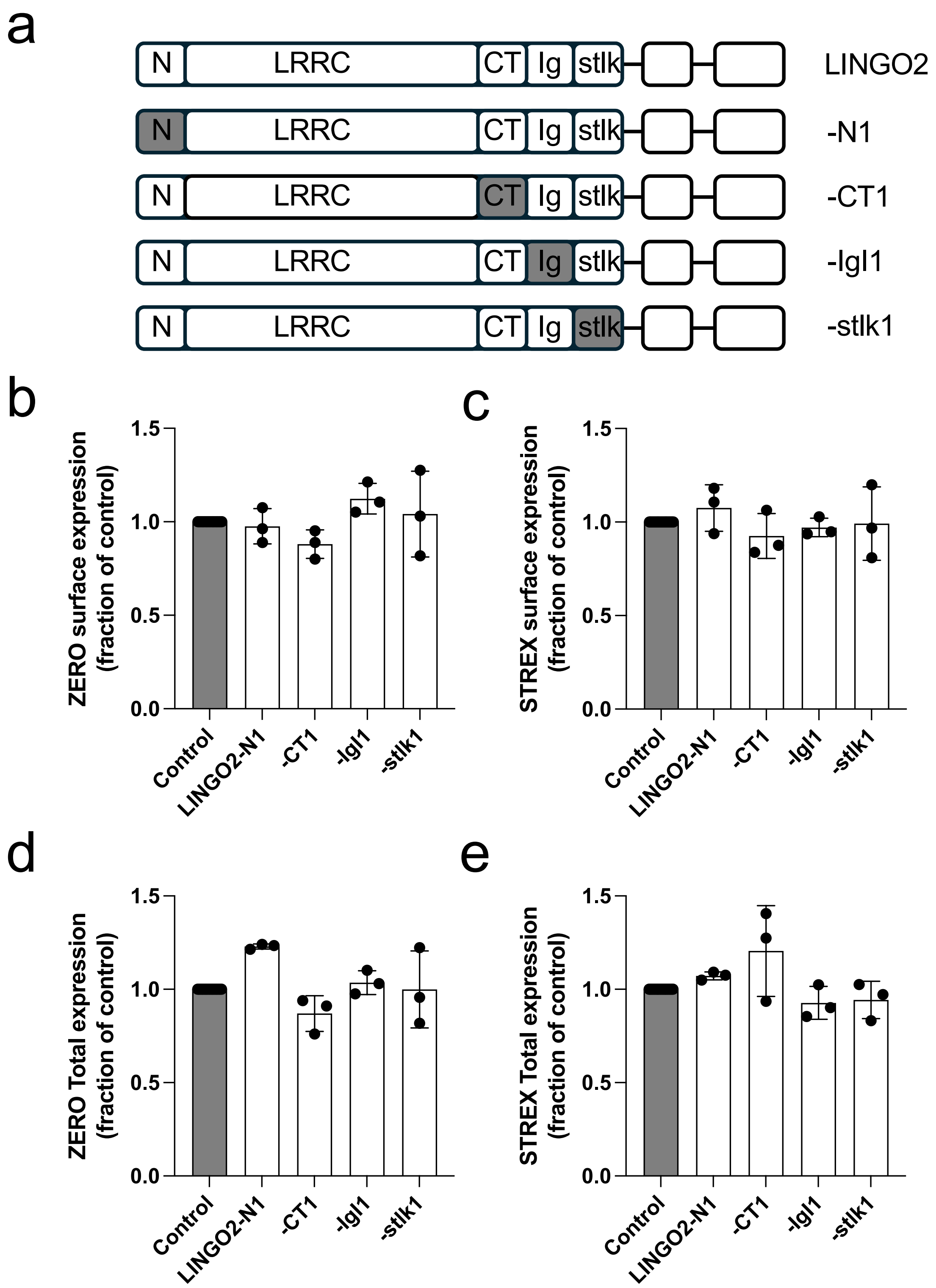
a
N
LRRC
CT
Ig
stlk
LINGO2
-N1
-CT1
-IgI1
-stlk1
b
ZERO surface expression (fraction of control)
c
STREX surface expression (fraction of control)
d
ZERO Total expression (fraction of control)
e
STREX Total expression (fraction of control)
0.0
0.5
1.0
1.5
Control
LINGO2-N1
-CT1
-IgI1
-stlk1

Figure S7

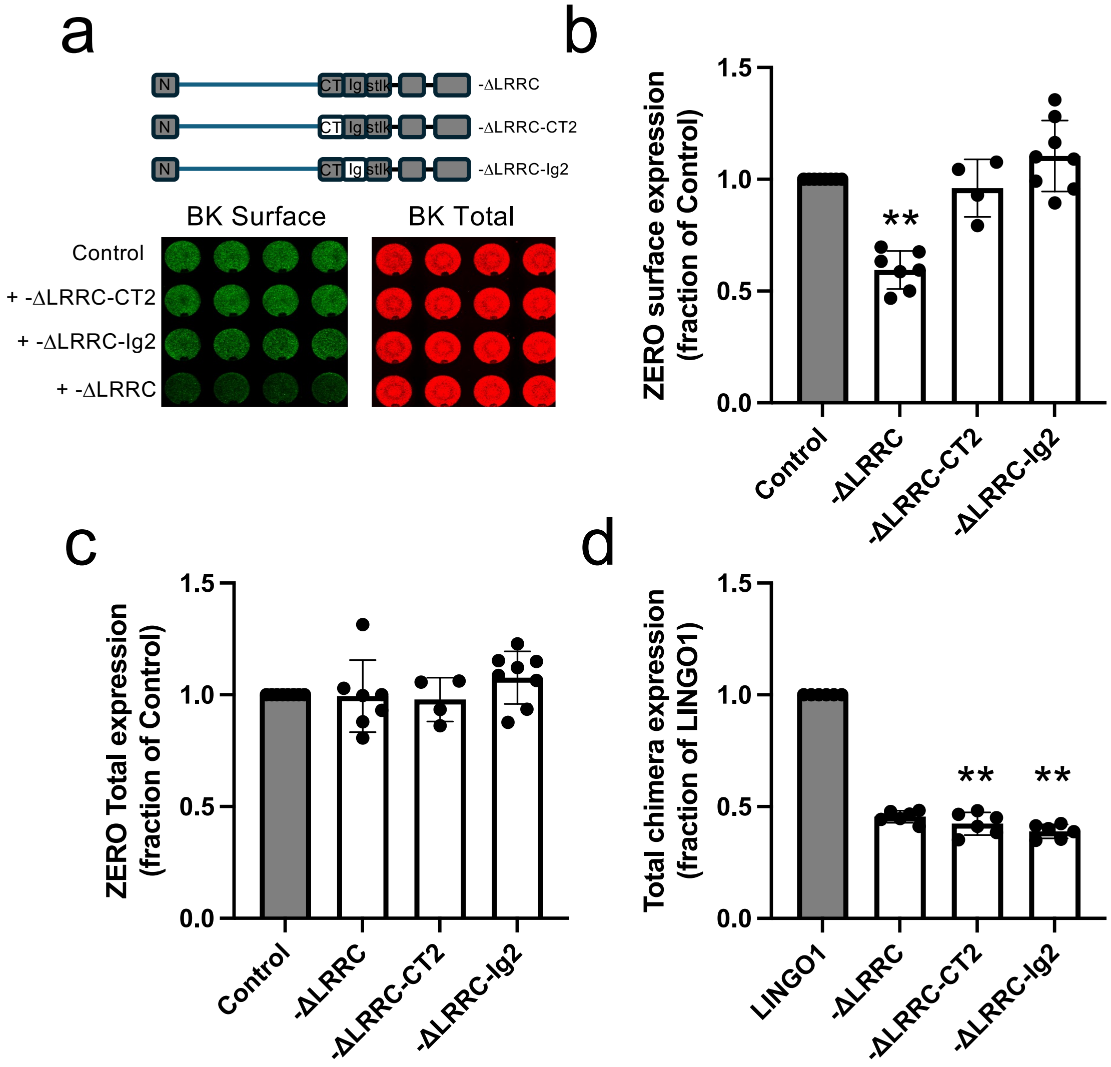

a
N
CT
Ig
stlk
-ΔLRRC
-ΔLRRC-CT2
-ΔLRRC-Ig2
BK Surface
BK Total
Control
+ -ΔLRRC-CT2
+ -ΔLRRC-Ig2
+ -ΔLRRC
b
ZERO surface expression
(fraction of Control)
1.5
1.0
0.5
0.0
**
Control
-ΔLRRC
-ΔLRRC-CT2
-ΔLRRC-Ig2
c
ZERO Total expression
(fraction of Control)
1.5
1.0
0.5
0.0
Control
-ΔLRRC
-ΔLRRC-CT2
-ΔLRRC-Ig2
d
Total chimera expression
(fraction of LINGO1)
1.5
1.0
0.5
0.0
**
**
LINGO1
-ΔLRRC
-ΔLRRC-CT2
-ΔLRRC-Ig2

Figure S8

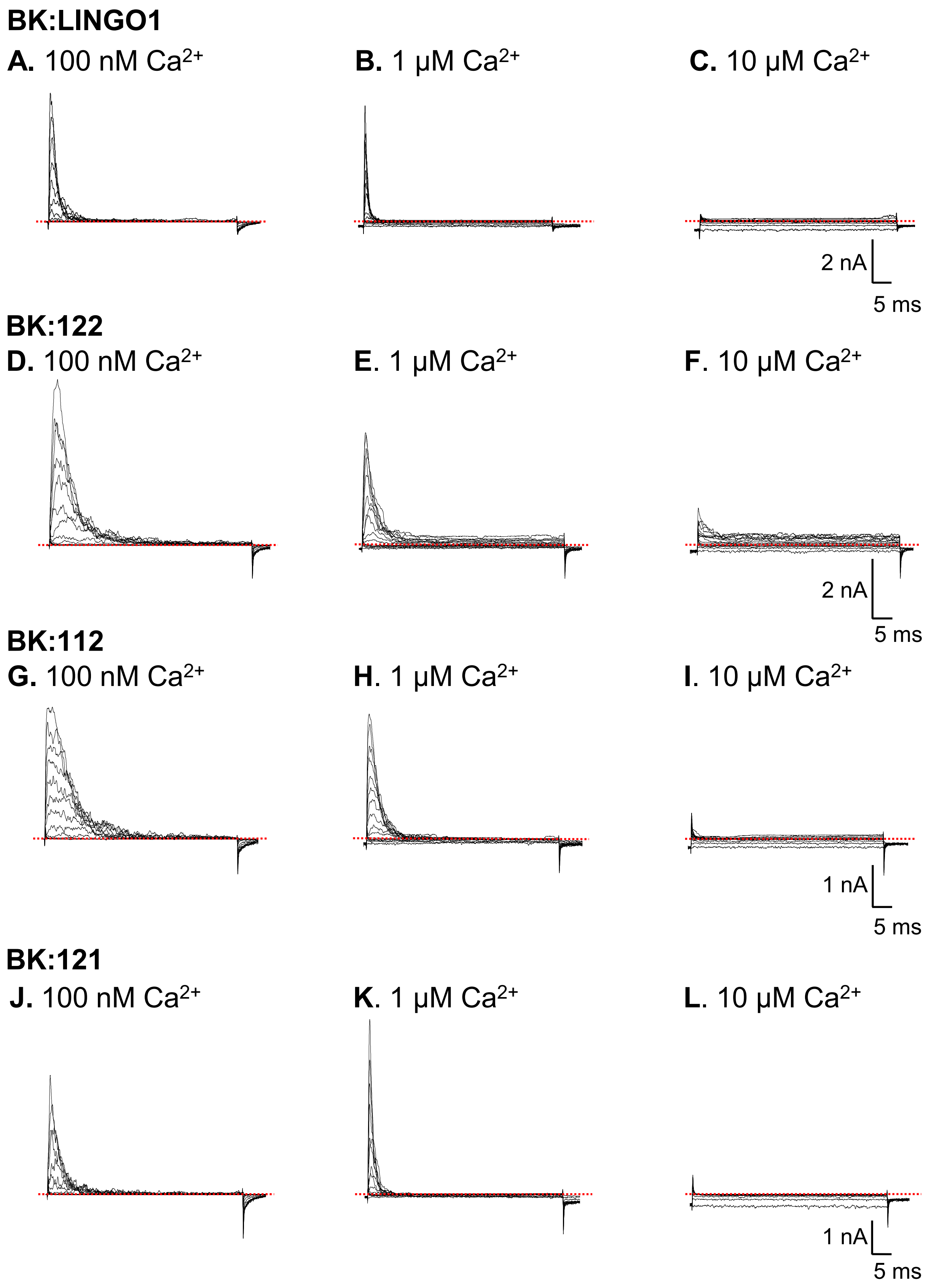
BK:LINGO1
A. 100 nM Ca2+
B. 1 µM Ca2+
C. 10 µM Ca2+
2 nA
5 ms
BK:122
D. 100 nM Ca2+
E. 1 µM Ca2+
F. 10 µM Ca2+
2 nA
5 ms
BK:112
G. 100 nM Ca2+
H. 1 µM Ca2+
I. 10 µM Ca2+
1 nA
5 ms
BK:121
J. 100 nM Ca2+
K. 1 µM Ca2+
L. 10 µM Ca2+
1 nA
5 ms

Figure S9

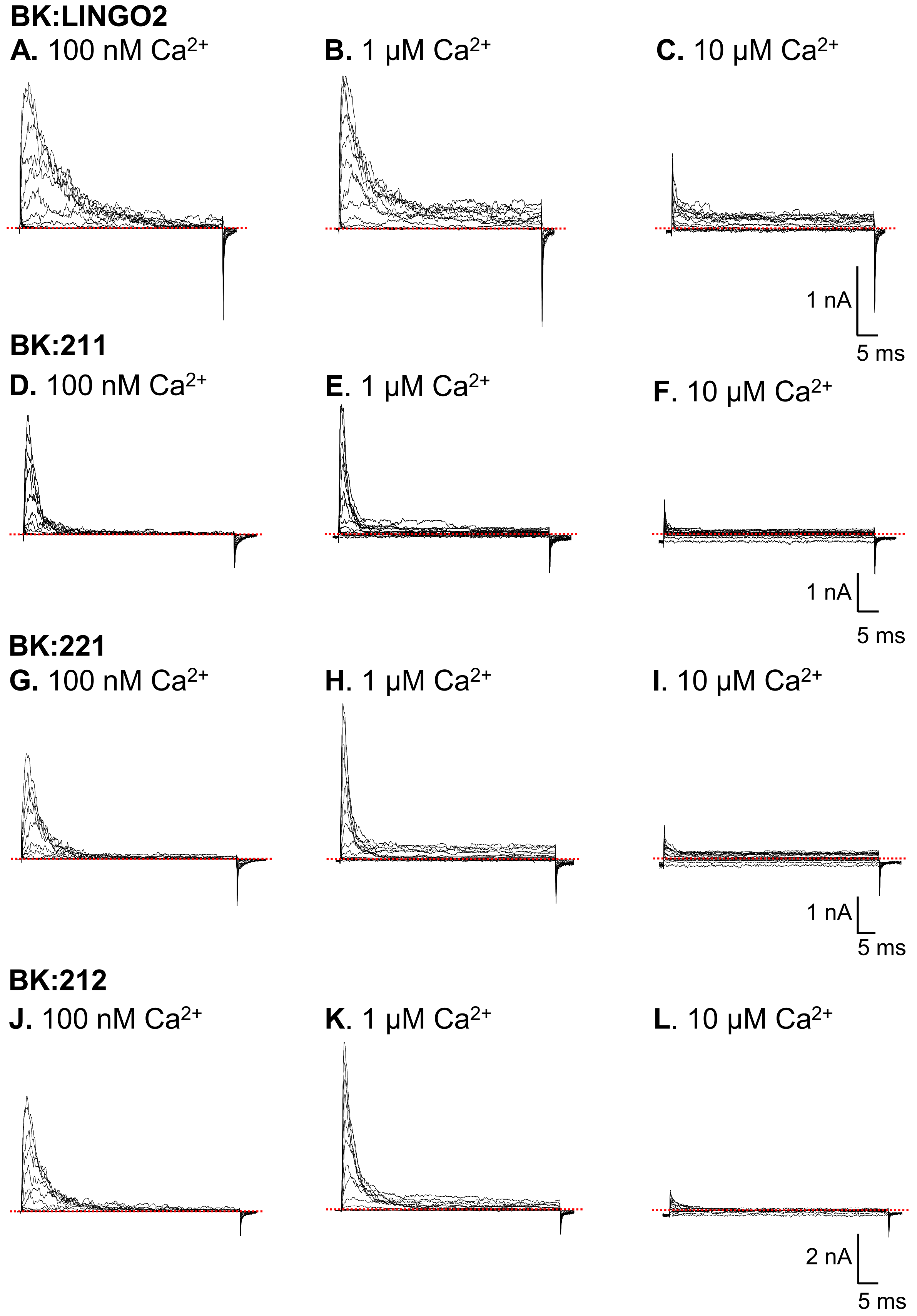
BK:LINGO2
A. 100 nM Ca2+
B. 1 μM Ca2+
C. 10 μM Ca2+
1 nA
5 ms
BK:211
D. 100 nM Ca2+
E. 1 μM Ca2+
F. 10 μM Ca2+
1 nA
5 ms
BK:221
G. 100 nM Ca2+
H. 1 μM Ca2+
I. 10 μM Ca2+
1 nA
5 ms
BK:212
J. 100 nM Ca2+
K. 1 μM Ca2+
L. 10 μM Ca2+
2 nA
5 ms

Figure S10

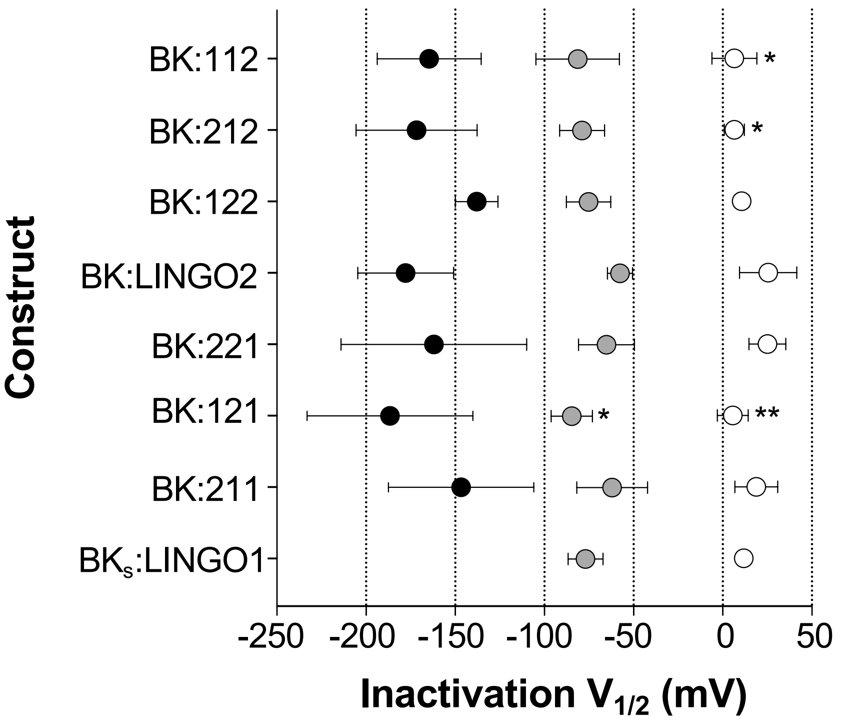
Construct
BK:112
BK:212
BK:122
BK:LINGO2
BK:221
BK:121
BK:211
BKs:LINGO1
*
*
*
**
-250
-200
-150
-100
-50
0
50
Inactivation V1/2 (mV)

Supplementary Figure Legends

Figure S1: *Sequence alignment of LINGO1 and LINGO2 domains.*

Amino acid sequence alignment of LINGO1 and LINGO2 indicating the extracellular domain (ED), transmembrane domain (TMD) and intracellular tail domain (ITD). The site of insertion of the -HA tag (..YPYDVPDYA..) epitope tag in LINGO1and LINGO2 is indicated. The extracellular domain comprises an N-terminal domain (region shown is the domain swap used), 12 LRR repeats of the LRRC domain, the LRR-CT domain, Ig-like domain and stalk domain.

Figure S2: *LINGO1 inhibits BK channel surface expression in a concentration dependent manner.*

OCW assays using fixed amounts (1μg) of the Flag- and -myc tagged ZERO or STREX variant of BKα with varying amounts of LINGO1 or LINGO2 expressed in HEK293 cells as in Figure 1. Surface expression of **A)** ZERO and **B)** STREX channels with LINGO1 (filled circles) or LINGO2 (open circles) with surface expression expressed as a fraction of the corresponding ZERO or STREX channel in the absence of LINGO1 or LINGO2. The corresponding total **C)** ZERO and **D)** STREX channel expression under each condition. Data are mean ± SD, n = 8/group for LINGO1 and n=5/group for LINGO2. * $p<0.05$ and ** $p<0.01$ compared to BKα alone, Kruskal-Wallis with Dunn's post-hoc test.

Figure S3: *Total expression of LINGO chimeras expressed in HEK293 cells*

A) Total expression of LINGO1 and LINGO2 and their respective extracellular domain (ED), transmembrane domain (TMD) or intracellular tail domain (ITD) chimeras expressed in HEK293 cells as in Figure 1. **B)** Total expression of LRRC repeat deletion (ΔLRRC) and sub-domain swaps in the ED of LINGO1 with corresponding LINGO2 subdomains as in Figure 2 **C)** Total expression of sub-domain swaps in the ED of LINGO2 with corresponding LINGO1 subdomains as in Figure S5. Data are mean ± SD from 3-9 experiments in each group. *

p<0.05, ** p<0.01 vs Lingo1 alone in panels A & B or vs LINGO2 alone in panel C, Kruskal-Wallis with post hoc Dunn's test.

Figure S4: *LINGO1 does not affect internalisation of BK channels.*

A) Schematic of internalisation assay using Flag- tag of epitope tagged ZERO and STREX channels to probe for surface channel (at time = 0) and 60 mins after internalisation at 37°C (t = 60). **B)** Representative experiment with 4 technical replicates showing surface (t = 0) and internalised (t = 60) Flag- tag labelling of BK channel in the presence and absence of LINGO1. Mock transfected (empty plasmid) HEK293 are also shown. **C)** Summary bar chart of internalisation of BK channel in the presence of LINGO1 expressed as a fraction of internalisation of BK channels in the absence of LINGO1. Data are mean ± SD, n=4 independent experiments per group.

Figure S5: *Lingo1 ΔLRRC chimera retains inactivation of BK channels.*

A) Schematic of LINGO1 ΔLRRC chimera in which the 12 LRR repeats in the ED are deleted. **B)** Representative inactivating currents in inside out patches in the presence of 100 nM Ca^{2+}. **C)** G/V plot of BK: ΔLRRC currents in the presence of 100 nM Ca^{2+}. Data are mean ± SD from 9 independent cells.

Figure S6 *Individual LINGO1 ED sub-domain swaps into LINGO2 ED domain do not confer inhibition of BK channel surface expression on LINGO2*

A) Schematic of LINGO chimeras with sub-domain swaps in the ED of LINGO2 with corresponding LINGO1 subdomains: N-terminus (N-), C-terminal LRR-CT (-CT), Ig-like domain (Ig1), stalk domain (-stalk) **B) & C)** Quantification of epitope tagged Flag-ZERO-myc and Flag-STREX-myc splice variant of BK channel surface expression with different LINGO2 ED sub-domain chimeras, expressed as a fraction of the Flag/myc ratio in the absence of LINGO2. **D) & E)** corresponding total BK channel expression expressed as a fraction of total

BK channel in absence of LINGO2. Data are mean ± SD from 3-7 independent experiments in each group.

Figure S7: *Individual LINGO2 ED sub-domain swaps in LINGO1-ΔLRRC rescue surface expression of ZERO BK channels*

A) Schematic of LINGO1- ΔLRRC chimeras with sub-domain swaps in the ED of LINGO1- ΔLRRC with corresponding LINGO2 subdomains: C-terminal LRR-CT (-CT2) or Ig-like domain (-Ig2). Representative experiment from an OCW assay to detect cell surface and total expression of the Flag-ZERO-myc variant of BK channels in HEK293 cells in the presence of the different LINGO- ΔLRRC chimeras, run in quadruplicate. **B)** Quantification of BK channel surface expression for ZERO and STREX splice variant of BK channel, expressed as a fraction of the Flag/myc ratio in the absence of LINGO1. **C)** corresponding total BK channel expression expressed as a fraction of total BK channel in absence of LINGO1. **D)** Total LINGO1−ΔLRRC chimera expression expressed as a fraction of LINGO1. Data are mean ± SD from 6-9 experiments in each group. * $p<0.05$, ** $p<0.01$ vs BK alone (Control) in panel B or vs LINGO1 alone in panel D, Kruskal-Wallis with post hoc Dunn's test.

Figure S8: *Inactivating BK currents of LINGO1 ED chimeras at different* Ca^{2+}.

Representative BK:LINGO chimera currents in the presence of 100 nM, 1 μM and 10 μM Ca^{2+} in isolated inside-out patches from HEK293 cells co-expressing BKα and LINGO1 (**A-C**), chimera 122 (**D-F**), chimera 112 (**G-I**) and chimera 121 (**J-L**).

Figure S9: *Inactivating BK currents of LINGO2 ED chimeras at different* Ca^{2+}.

Representative BK:LINGO chimera currents in the presence of 100 nM, 1 μM and 10 μM Ca^{2+} in isolated inside-out patches from HEK293 cells co-expressing BKα and LINGO2 (**A-C**), chimera 211 (**D-F**), chimera 221 (**G-I**) and chimera 212 (**J-L**).

Figure S10: *Comparison of steady state voltage-dependent inactivation properties of LINGO1, LINGO2 and their chimeras.*

Experiments were carried out in the presence of 100 nM Ca^{2+} (white symbols), 1 μM Ca^{2+} (grey symbols) and 10 μM Ca^{2+} (black symbols). Data shows mean ± SD for between 4 and 15 patches in each chimera and [Ca^{2+}]. All experiments were carried out on rabbit BK, with the exception of the BK_S:LINGO1 experiments which utilized the mouse STREX variant. The inactivation $V_{1/2}$ was compared against BK:LINGO2 in each of the three [Ca^{2+}] in each construct. * $p<0.05$ and ** $p<0.01$ compared to BK:LINGO2, one-way ANOVA, Dunnett's multiple comparison test.
